# A genetics‐based approach confirms immune associations with life history across multiple populations of an aquatic vertebrate (*Gasterosteus aculeatus*)

**DOI:** 10.1111/mec.14772

**Published:** 2018-07-09

**Authors:** James R. Whiting, Isabel S. Magalhaes, Abdul R. Singkam, Shaun Robertson, Daniele D'Agostino, Janette E. Bradley, Andrew D. C. MacColl

**Affiliations:** ^1^ School of Life Sciences University of Nottingham University Park, Nottingham UK; ^2^ School of Life Sciences University of Sussex Falmer, Brighton UK; ^3^ Department of Life Sciences Whitelands College University of Roehampton London UK; ^4^ Pendidikan Biologi JPMIPA FKIP University of Bengkulu Bengkulu Indonesia; ^5^ Institute of Biodiversity, Animal Health and Comparative Medicine University of Glasgow Glasgow UK

**Keywords:** adaptation, ecoimmunology, immune variation, life history evolution, population genetics, senescence

## Abstract

Understanding how wild immune variation covaries with other traits can reveal how costs and trade‐offs shape immune evolution in the wild. Divergent life history strategies may increase or alleviate immune costs, helping shape immune variation in a consistent, testable way. Contrasting hypotheses suggest that shorter life histories may alleviate costs by offsetting them against increased mortality, or increase the effect of costs if immune responses are traded off against development or reproduction. We investigated the evolutionary relationship between life history and immune responses within an island radiation of three‐spined stickleback, with discrete populations of varying life histories and parasitism. We sampled two short‐lived, two long‐lived and an anadromous population using qPCR to quantify current immune profile and RAD‐seq data to study the distribution of immune variants within our assay genes and across the genome. Short‐lived populations exhibited significantly increased expression of all assay genes, which was accompanied by a strong association with population‐level variation in local alleles and divergence in a gene that may be involved in complement pathways. In addition, divergence around the *eda* gene in anadromous fish is likely associated with increased inflammation. A wider analysis of 15 populations across the island revealed that immune genes across the genome show evidence of having diverged alongside life history strategies. Parasitism and reproductive investment were also important sources of variation for expression, highlighting the caution required when assaying immune responses in the wild. These results provide strong, gene‐based support for current hypotheses linking life history and immune variation across multiple populations of a vertebrate model.

## 1 INTRODUCTION

Parasitism is ubiquitous in nature (Poulin & Morand, 2000; Windsor, 1998), and variation in immune responses amongst wild populations is extensive at the phenotypic and genotypic level (Martin, Hawley, & Ardia, 2011; Pedersen & Babayan, 2011). This variation exists because natural environments expose costs of immune responses such as energy expenditure (Schmid‐Hempel, 2011), autoimmunity (Graham, 2002; Graham, Allen, & Read, 2005; Kopp & Medzhitov, 2009) and antagonisms between responses to different kinds of parasites (Kamath, Turner, Küsters, & Getz, 2014; Loiseau et al., 2008). Immune variation likely has implications for host fitness; therefore, the identification of correlated variation in other traits will help identify the ultimate causes of variation.

The pace‐of‐life‐syndrome concept (Reale et al., 2010) suggests that a suite of physiological traits, such as metabolism, hormones and immunity, have coevolved alongside population‐level life history variation. The idea suggests that given ecological conditions favouring a particular life history, where life history can vary along a continuum between traditional r (short‐lived, rapid growth, many low‐cost offspring, high mortality) and K (long‐lived, long development, few high‐cost offspring, low mortality) strategies (Promislow & Harvey, 1990; Ricklefs & Wikelski, 2002), other traits including immune responses should vary predictably. However, there remain gaps in the empirical literature supporting a relationship between life history and immune responses.

There are contrasting theories regarding the nature of the relationship between immune responses and life history. Lee (2006) argues that short‐lived species should offset the costs of immune phenotypes through increased extrinsic mortality and thus should adopt developmentally cheap but usage‐costly immune defences that are associated with autoimmunity and are more energetically demanding, such as innate inflammatory and Th1‐mediated responses. Longer‐lived species conversely should invest more in specific, less damaging and energetically cheaper adaptive responses (Lee, 2006). Alternatively, shorter life history strategies may favour an absence of immune investment in general, resulting in weaker responses as resources are partitioned into life history traits, such as rapid development and reproduction (Norris & Evans, 2000). This has been observed in *Daphnia magna*, whereby the presence of predatory fish kairomones increases allocation into growth and reproduction, which subsequently increases susceptibility to the rotifer parasite *Brachionus rubens* (Pauwels, De Meester, Michels, Jeppesen, & Decaestecker, 2014). Disagreement between these theories likely stems from how immune responses are defined, either specifically with associated costs or at a general level of immunocompetence.

Studies of closely related species appear to support the theories of Lee (2006), with empirical evidence existing from a diverse range of taxa; including birds (Lee, Wikelski, Robinson, Robinson, & Klasing, 2008; Pap et al., 2015; Tella, Scheuerlein, & Ricklefs, 2002; Tieleman, Williams, Ricklefs, & Klasing, 2005), mammals (Previtali et al., 2012) and even corals (Pinzon, Dornberger, Beach‐Letendre, Weil, & Mydlarz, 2014). However, there are examples in which life history–immune relationships are weak or contrary at the species level (Horrocks et al., 2015; Martin, Weil, & Nelson, 2007; Matson, Cohen, Klasing, Ricklefs, & Scheuerlein, 2006). It therefore remains unclear how these relationships evolve in nature. Such disagreement may stem from a failure to account for evolutionary history. For example, Bech, Chappell, Astheimer, Londoño, and Buttemer (2016) note that ancestral phenotypes explain indistinguishable basal metabolic rates (BMR) between sympatrically occurring short‐ and long‐lived Australian passerines, despite the expectation of a negative association.

Confounding evolutionary history complicates inferences drawn from between‐species comparisons, and therefore, within‐species variation may be more informative. However, individual‐ or population‐level analyses are less frequent in the literature, (but see Rantala & Roff, 2005; Sparkman & Palacios, 2009). In addition, few studies have examined life history–immune associations from a genetic perspective. The consensus has generally been to use biomarkers and immune assays to quantify an immune phenotype, but such measures can be strongly susceptible to plasticity in the wild (Galli, Borregaard, & Wynn, 2011; Luoma, Butler, & Stahlschmidt, 2016). This risks leading to alternative explanations for life history associations (Palacios, Cunnick, & Bronikowski, 2013) and variable conclusions being drawn depending on the marker sampled (Christensen et al., 2016). From an evolutionary perspective, this empirical gap is particularly concerning, and linking heritable, genetically determined immune variation to life history within species remains a challenge to overcome to understand wild immune variation.

There therefore exist two barriers to empirically examining the evolutionary relationships between life history and immune variation. First, evolutionary history needs to be accounted for and we do this by examining populations within a species, the three‐spined stickleback, *Gasterosteus aculeatus*, with contrasting life history strategies. Second, when dealing with wild individuals the issue of unknown plasticity must be considered. We do this is in two ways with an emphasis on genetic variation in immune genes. First, we focus on immune gene expression assays in adult males, including measures of reproductive condition, which are likely to have comparable phenotypes. As male stickleback overwhelmingly have a single breeding season and perform all parental care, incurring costs associated with nest‐building, territoriality, ornamentation and fry rearing (Whoriskey & Fitzgerald, 1994), fish displaying reproductive traits are likely to be stressed and verging on senescent. Stressed individuals within a population should reveal resource‐based plastic effects on the immune response. We assess how immune gene expression varies with measures of reproductive condition to estimate what constitutes a “cheaper” response in this system, which in line with predictions may manifest as shifts between types of immune response and/or more general changes in immune activity. This approach is also informative about flexibility of immune gene expression within populations (when genetic variation is minimal) and where caution must be taken when interpreting results between populations. With defined “cheaper” responses, we can interpret variation between life history strategies highlighted in predictions outlined above. Specifically, we expect short‐lived populations to evolve either costly immune responses (in keeping with the hypothesis that higher extrinsic mortality offsets costs) or less costly responses (in line with the hypothesis that immune responses are traded off against rapid growth and reproduction).

Second, to draw evolutionary conclusions, because gene expression is itself subject to plasticity, we compare our measures of population‐level variation in immune gene expression to a genetic approach based on SNPs in immune‐related genes. To understand the genetic basis for any expression variation, we assess the allelic diversity of SNPs associated in and around our assay genes, to target coding sequence changes or changes to cis‐regulatory elements. We also incorporate a GWAS approach to look for immune‐linked genes across the genome that may have diverged between short‐lived and long‐lived populations. Finally, we expand the analysis to a larger group of populations and assess the comparative relationship between life history strategy and genetic variation around a large number of immune genes across the genome. Such an approach introduces a novel level of genetic accountability for immune covariance with life history that will help reveal the potential for evolutionary constraints or associations to occur.

Three‐spined stickleback represent an excellent study system for questions of evolutionary biology due to their rapid and repeated adaptive radiations from marine to diverse freshwater forms. Still extant marine populations are commonly assumed to be a good representation of the ancestor of freshwater populations which diverged following colonization from the sea ~10,000 years ago (Colosimo, 2005). Diverged freshwater stickleback exhibit strong life history variation in keeping with known short‐lived and longer‐lived strategies (DeFaveri & Merilä, 2013; Gambling & Reimchen, 2012; Magalhaes, D'Agostino, Hohenlohe, & MacColl, 2016). Stickleback are also hosts to a plethora of parasites (De Roij & MacColl, 2012; MacColl, 2009; Poulin, Blanar, Thieltges, & Marcogliese, 2011; Young & MacColl, 2017), which have helped drive immune variation between wild freshwater populations (De Roij, Harris, & MacColl, 2011; Robertson, Bradley, & MacColl, 2016a,b; Scharsack, Kalbe, Harrod, & Rauch, 2007). This system therefore allows us to ask questions about covariation between life history and immune phenotypes across multiple populations within a single radiation, promoting our understanding of how life history and immune responses diverge alongside one another. To do this, we assessed fish from two short‐lived populations, two long‐lived populations and a proxy “ancestral” anadromous population to demonstrate how populations have diverged from the presumed ancestral immune phenotype. Anadromous stickleback are larger and typically reach sexual maturation after 2 years rather than one as in our four freshwater populations. This may suggest that the anadromous population has the “longest” life history; however, such an interpretation is potentially confounded by the addition of migration between marine and saltwater inland lochs.

## 2 MATERIALS AND METHODS

### 2.1 Study site and sample collection

Three‐spined stickleback were collected from the island of North Uist, Scotland, in April and May 2015. Fish were caught in unbaited Gee's Minnow Traps, set overnight in 5 lakes (lochs) (Table 1) that harbour populations with varying life histories. Fish in TORM and BHAR are mostly annual, but fish in REIV and HOSTA can live as long as 3 years and often survive for two breeding seasons (Rahman, 2017). Anadromous OBSM fish are largely 2 years old when they breed. We selected males from contrasting life stages from HOSTA and REIV (18 of each from both lochs, and only mature males from lochs BHAR (21), OBSM (23) and TORM (22), yielding a total of 138 fish. Life stage was assessed based on sexual maturity according to testis and kidney condition and sexual ornamentation according to characteristic nuptial coloration (carotenoid‐based red‐throat ornamentation). Fish were transported back to the laboratory in darkened boxes and sampled in a random order within 6 hr of collection. Sampling order was found to have no effect on results (Supporting Information).

**Table 1:** Sampling information for North Uist lochs. All lochs were sampled across April–May 2015

Code	Population name	Life History	Area	Latitude	Longitude	Sampled *N*
BHAR	a'Bharpa	Short	SE	57°34″N	7°17″W	21
HOST	Hosta	Long	NW	57°37″N	7°29″W	36
OBSM	Obissary	Anadromous	E	57°36″N	7°10″W	23
REIV	naReival	Long	W	57°37″N	7°31″W	36
TORM	Tormasad	Short	S	57°33″N	7°19″W	22

Fish were euthanized by MS222 overdose followed by destruction of the brain (in accordance with schedule 1 techniques described by UK Home Office regulations). Fish were then weighed and measured, before having their spleen removed and placed in RNAlater (Life Technologies). Samples of spleen, which is an important immune tissue in fish (Zapata, Diez, Cejalvo, Gutiérrez‐De Frías, & Cortés, 2006), were kept at 4°C for 24 hr and then at −20°C with RNA being extracted within 3 months. Reproductive condition was scored according to a qualitative scale based on size of testes and kidney (the kidney of breeding male stickleback becomes enlarged); 1 (small testes and kidney); 2 (enlarged, melanized testes, small kidney); 3 (enlarged, melanized testes and enlarged kidneys). Sexually mature males were also inspected for breeding coloration (characteristic red throat and blue eyes) and recorded as either displaying or not in a binary measure. These two measures of reproductive investment (qualitative score and breeding coloration) were handled separately in subsequent analyses. Testes and adipose tissue were removed and weighed. Somatic weight was calculated by subtracting testes and adipose tissue weight from total weight. This value was then used to calculated gonadosomatic index (GSI) and adiposomatic index (ASI) according to testes, or adipose weight/somatic weight × 100. Otoliths were extracted to estimate fish age and were stored in 100% ethanol before being mounted on slides and photographed under a light microscope. Age (in years) was estimated from otoliths (Jones & Hynes, 1950).

Macroparasite abundance for each fish was recorded for the ectoparasitic monogenean trematode *Gyrodactylus arcuatus* and the cestode *Schistocephalus solidus*, which are both commonly occurring and have well‐studied effects on host fitness (Barber & Scharsack, 2010; Barber, Wright, Arnott, & Wootton, 2008; De Roij et al., 2011; MacNab, Scott, Katsiadaki, & Barber, 2011; Rushbrook & Barber, 2006). All plerocercoids found in fish with *S. solidus* infections were also weighed, and a Schistosomatic Index (SSI) was calculated as above.

### 2.2 RNA isolation and qPCR

All qPCR work was conducted in accordance with the MIQE guidelines (Bustin et al., 2009). Sampling order was randomized, and RNA was extracted from stored, whole spleens using the Genejet RNA purification kit (Thermo Scientific) according to the manufacturer's protocol. RNA was DNase treated using Precision DNase (Primerdesign) following the manufacturer's protocol.

RNA purity was assessed on a NanoDrop1000 spectrophotometer (Thermo Scientific) blanked using DNase (Primerdesign)‐treated nuclease‐free water incubated under equivalent conditions. RNA integrity was assessed following DNase treatment by visualization of 4 μl of sample on a 2% agarose gel stained with ethidium bromide. Reverse transcription was performed using nanoScript2 RT kit (Primerdesign) according to the manufacturer's protocol using approximately 1.5 μg of template. This protocol uses a combination of random nonamer and oligo‐dT priming. Genomic DNA contamination was assessed via light PCR using intron‐spanning primers. All cDNA samples were diluted 1:10 with nuclease‐free water before use.

A total reaction volume of 10 μl was used to perform qPCR reactions consisting of 5 μl of PrecisionFAST low ROX mastermix with SYBR green (Primerdesign), 2.5 μl of nuclease‐free water, 0.25 μl of each primer at working concentration and 2 μl of cDNA template. Reactions were performed in 96‐well optical PCR plates with optical seals (StarLab) in an ABI 7500 Fast real‐time thermocycler (Applied Biosystems). For all assays, samples were incubated at 95°C for 20 s, followed by 45 cycles of 95°C for 3 s and 60°C for 30 s. A melt curve analysis was also included to confirm product.

### 2.3 Choice of assay genes

Genes for which assays had been previously developed (Robertson, Bradley, & MacColl, 2016a) were selected to characterize different arms of the immune response and were identified based on previous studies in other fish and known roles of orthologous genes. The proinflammatory gene *tnf*α is a key component of innate immunity in teleosts and other vertebrates (Secombes & Wang, 2012; Uribe, Folch, Enriquez, & Moran, 2011), activating macrophages, eliciting inflammation and increased respiratory burst activity. The Th1 transcription factor *stat4* promotes the differentiation of Th1 cells and has been identified in teleost genomes. Expression of Th1‐associated genes in infection studies suggests that fish may possess a full and conserved Th1 pathway (Secombes & Wang, 2012). Th1 adaptive immunity is associated with intracellular parasites, whilst Th2 adaptive immunity is associated with extracellular infection. Th2 cell differentiation is associated with upregulation of *cmip* and *stat6* in mammals, which is also upregulated alongside other markers of Th2 responses in zebrafish head kidney and spleen cells in response to immunostimulation (Mitra, Alnabulsi, Secombes, & Bird, 2010). Finally, *foxp3a* expression characterizes the regulation of immune responses by Treg cells. This gene has been identified in many teleost genomes and has demonstrable roles in immune regulation in fish and mammals (Secombes & Wang, 2012). The genes included in this study therefore serve to capture immune variation in innate (*tnf*α), Th1 adaptive (*stat4*), Th2 adaptive immunity (*cmip, stat6*) and Treg regulation (*foxp3a*).

### 2.4 Gene expression quantification

A custom stickleback geNorm analysis with SYBR green was conducted to select appropriate housekeeping genes for this experiment. The analysis was conducted according to the manufacturer's protocol (Primerdesign) using 15 randomly selected samples made up of 2/3 fish from each population. Of the six candidate reference genes supplied (*b2m*,* gapdh*,* rpl13a*,* hprt1*,* tbp* and *top1*), *b2m* and *hprt1* were the most stably expressed combination.

In total, 138 spleen samples were used from five populations. A reference sample, comprised of a pool sample of all individuals, was made up and used as a control reference across all plates. Samples were randomly assigned plates with 46 duplicated samples to a plate, along with a duplicated reference and negative controls. The five genes included in this study were amplified using primers published in Robertson, Bradley, and MacColl (2016a) (Table 2).

**Table 2:** Primer details for qPCR assays

Gene	ENSEMBL ID	Immune Role	Primer Sequence (5′‐3′)	Amplicon length
*tnf*α	ENSGACG00000013372	Proinflammatory cytokine	Fwd‐GCTTGGTTCTGGCCAGGTTT	125
			Rev‐GCTGCTGATTTGCCTCAACG	
*stat4*	ENSGACG00000002684	Transcription factor for differentiation of Th1 cells	Fwd‐CTCTCAGTTTCGAGGCTTGCTT	100
			Rev‐GGCAGTTGGCTCACATTGG	
*cmip*	ENSGACG00000002527	Signalling protein in Th2 pathway	Fwd‐GGCATGGAGGTCGTCAAGAA	119
			Rev‐TAGCAGGAGTAAATGGCGGC	
*stat6*	ENSGACG00000008477	Involved in mediating Th2 cytokines IL‐4 and IL‐3 signalling	Fwd‐CTCAGCCACAGTTCCAACCGTTC	104
			Rev‐GTCGGATGTTCTGGACCTCGAGT	
*foxp3a*	ENSGACG00000012777	Promotes development and function of Treg cells	Fwd‐GTTGACCCATGCAATTCCGA	94
			Rev‐CTGCTGTAGTTGTGGTCCTG	

Relative expression ratios were calculated according to the ΔΔ*C*_q_ method (Pfaffl, 2001) and adjusted for the amplification efficiencies of each primer pair. Expression values were standardized against the geometric mean Cq of two reference genes, selected via a GeNorm kit (Primerdesign) according to the manufacturer's protocol.

### 2.5 Data analysis

All data were analysed in R (version 3.3.1), and data for the 138 individuals were subsetted into two analyses. The first subset comprised all males from lochs HOSTA and REIV, totalling 71 individuals. The second subset included mature males from all lochs, totalling 102 individuals.

For each subset, the following analysis was conducted. Log^10^‐transformed relative expression values were assessed for covariance in a principal component analysis. PC1 and PC2 scores were then modelled as dependent variables in generalized linear models (GLM). For the HOSTA‐REIV subset, these were modelled with the independent variables: sampling loch (LOCH), qualitative reproductive score (REPRO), breeding coloration (0 or 1, RED), age (AGE), *Gyrodactylus* load (GYRO) and infection status (0 or 1, GYRO_X), schistosomatic index (SSI) and infection status (SCHISTO_X), gonadosomatic index (GSI), adiposomatic index (ASI) and length standardized by population mean (LENGTH). ASI was also modelled using a Gamma family and log link by the other independent variables to clarify the assumption that qualitative reproductive score, as a proxy for breeding, is negatively associated with condition.

Expression data from the subset of all lochs were modelled in linear mixed models (LMMs) using the same independent variables apart from RED (as almost all individuals in this subset were in breeding coloration). Here, however, fish were categorized by life history strategy (LH), with LOCH retained as a random effect to account for multiple lochs per LH. Variables were modelled additively and removed sequentially using a top‐down approach. The model with the best fit, assessed using the Akaike information criterion (AIC), was then modified to include biologically plausible interactions between independent variables that may improve fit. If model fit was significantly improved, interactions were added, and if not, the simplest model was retained. Variable significance was assessed using anova and post hoc Tukey tests for categorical variables where appropriate.

### 2.6 RAD‐seq data handling

RAD sequence alignments were acquired for all five populations from those published in Magalhaes et al. (2016), with all lochs being sampled in Spring 2013. In total, 91 fish were included in this analysis, split between HOSTA (19), REIV (19), TORM (19), BHAR (18) and OBSM (16). The STACKS pipeline (Catchen, Hohenlohe, Bassham, Amores, & Cresko, 2013) was used to analyse mapping files, and population genetics statistics were calculated using the populations program in Stacks. The following filters were applied: SNPs that were not present in all populations were removed; SNPs present in <80% of individuals within a population were removed; SNPs with a minor allele frequency below 0.05 were removed; the first SNP of each RAD locus was retained to avoid linked loci; and data from the sex chromosome (XIX) were also removed. Following filtering, the final output consisted of 11,558 SNPs. Population genetics statistics were output to genepop format and converted to plink and arlequin formats using PGDSpider2 (Lischer & Excoffier, 2011).

### 2.7 Population structure analysis

plink files were used with faststructure (Raj, Stephens, & Pritchard, 2014) to assess genetic structure between the five populations under a Bayesian framework for posterior inference from large SNP genotype data. The algorithm was run for 1–5 populations (K) using faststructure's default conversion criterion of 10e‐6 and the simple prior. faststructure is packaged with a default algorithm for estimating maximum likelihood for K, which was used in this instance.

### 2.8 Outlier analysis

Outlier analysis was performed on the unlinked 11,558 SNPs spread across the genome using arlequin (Excoffier & Lischer, 2010) and bayenv2 (Günther & Coop, 2013). For arlequin, population structure was input using results from faststructure and loci were detected using 20,000 simulations with 100 demes per group, and a minimum and maximum expected heterozygosity of 0.1 and 1.0, respectively. Loci that exhibit higher or lower *F*_ST_ in comparison with a null distribution are deemed to be under either directional or balancing selection, respectively. *p*‐values were corrected for multiple testing using the R package *qvalue* (Storey, Bass, Dabney, & Robinson, 2015). To find loci under selection, arlequin uses the fdist2 method, which has been demonstrated to exhibit a high rate of false positives (Whitlock & Lotterhos, 2015). To account for this, we also used the X^T^X statistic packaged with bayenv2 to cross‐examine the data. The X^T^X statistic is analogous to *F*_ST_ and is a Bayesian measure of population differentiation. Population structure was accounted for using a covariance matrix calculated according to the author's instructions (Günther & Coop, 2013). bayenv2 was run independently five times over 100,000 iterations and final X^T^X values were averaged across runs. Loci that were deemed to be under selection had *p*‐values <0.05 (after FDR corrections) according to arlequin and were in the 95^th^ percentile of X^T^X scores according to bayenv2. SNPs were mapped back to genes by comparing location information within the alignment files to gene locations extracted from Ensembl's BioMart (Smedley et al., 2015) using a custom bash script. Gene Ontology (GO) was also inferred from BioMart.

### 2.9 Genetic variance of assay genes

To investigate genetic variability of our 5 qPCR assay genes, we repeated the populations analysis above with a subset of RAD‐loci found within the five genes or within 50‐kB flanking regions. Filtering conditions were also relaxed to increase the number of SNPs available: SNPs present in at least two of the five populations were retained; SNPs present in <50% of individuals were removed; minor allele frequency was maintained at 0.05; all SNPs within whitelisted loci were permitted. In total, this analysis used 51 SNPs across 18 RAD‐loci. PCAs were performed using *adegenet* (Jombart, 2008) in R and visualized using *ggplot2* (Wickham, 2016). PC1 and PC2 scores were extracted and averaged across populations; these were included in Pearson's correlation analyses against PC1 and PC2 population averages of relative gene expression. This analysis was performed using the “corr.test” function in the R package *psych* with “holm” multiple comparison corrections. The relative contributions of SNPs towards these correlative relationships were assessed by means of PC loadings from individual SNPs in the genetic variance PCA.

### 2.10 Comparative relationship of immune gene genetic variance and life history

To expand the analysis to comparative levels, we analysed genetic variation in and around genes annotated with the GO term “immune system process” (GO:0002376) and assessed the relationship with life history strategy across 15 diverged population. GO:0002376 is a parent term for all GO terms associated with immune responses and covers 389 genes with various immune functions in the stickleback genome (BROADs1, Ensembl release 90). RAD‐seq data for the additional 11 freshwater populations are the same as those published in Magalhaes et al. (2016) (Supporting Information Table S1). The following filters were applied: SNPs that were present in <8 populations were removed; SNPs present in <50% of individuals within a population were removed; SNPs with a minor allele frequency below 0.05 were removed; all SNPs were retained per locus; and data from the sex chromosome (XIX) were also removed. SNPs were further filtered as being within the 389 immune genes or within 10‐kb flanks. This flanking region was reduced to reflect the larger number of genes included here compared with our five assay genes. populations was run with the following filters: SNPs present in at least eight of the 15 populations were retained; SNPs present in <50% of individuals were removed; minor allele frequency was maintained at 0.05; all SNPs within whitelisted loci were permitted. After filtering, this analysis used 597 SNPs across 360 RAD‐loci, which captured variation around 166 of the 389 immune genes annotated in the genome (Supporting Information Table S2). PCAs were performed using adegenet (Jombart, 2008), with PC1 scores extracted for further analysis.

Life history data included per cent of wild population over 1 year old in breeding season; size (standard length) at 1 year of age; age at maturity; and size at maturity (Rahman, 2017). These data were compiled into a single PCA axis with PC1 scores for each loch. Neutral population structure for these populations was determined by pruning genomewide SNPs for linkage in PLINK (–indep‐pairwise 50 5 0.2) and performing PCA as above with the retained 15,662 SNPs. PC scores for PCs 1–10 were then averaged per fish with weightings according to each PC's eigenvalue. Weighted average PC scores were then averaged by population to reflect population‐level averages for life history variation.

Life history and neutral structure variables were used to model immune gene genetic variation PC1 scores using linear mixed models. A random factor of LOCH was included to account for multiple genetic variation PC1 and life history PC1 scores per population.

## 3 RESULTS

### 3.1 Relative expression of immune genes

#### 3.1.1 REIV and HOSTA (Males of contrasting sexual maturity)

**Figure 1:**
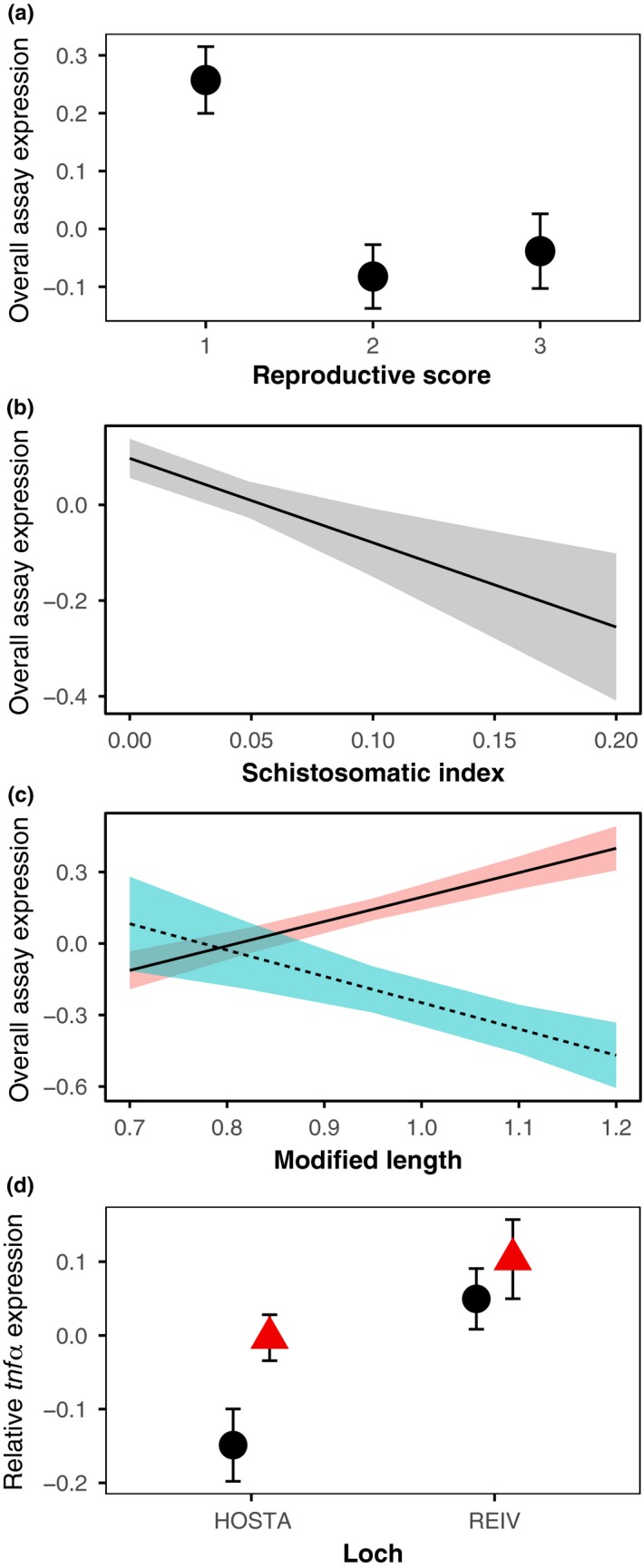
Significant factors in minimum adequate GLMs fitted to overall assay expression (PC1) scores (a–c) and relative *tnf*α expression (PC2) scores (d) for fish sampled from REIV and HOSTA. Effect plots (a–c) visualize the mean effect of an individual factor with standard error whilst other factors within the model are maintained at a constant. (a) The effect of reproductive condition on overall immune expression. (b) The effect of *S. solidus* infection intensity on overall immune expression. (c) The effect of the interaction between length (Modified Length) and *S. solidus* infection on expression of all immune genes. Line colour and type represent infection group; red solid line shows uninfected fish; blue dashed line shows infected fish. (d) The effect of sampling location and breeding coloration (circle = no coloration, triangle = red throat) on relative *tnf*α expression. Points here show group mean PC2 scores with standard error [Color figure can be viewed at http://www.wileyonlinelibrary.com]

For a summary of GLM results, see Table 3. In our HOSTA‐REIV comparison of adult, sexually immature and mature males, PC1 and PC2 represented 59.1% and 18.2%, respectively (77.3% cumulatively), of the total variation in relative expression across the five genes. PC1 represented covariance of all genes’ expression in the same direction, whilst variation in PC2 was driven by increased expression of the inflammatory gene *tnf*α and generally reduced expression of the other 4 genes (Supporting Information Table S3; Figure S1). These PCs were therefore summarized to signify relative expression of all genes (PC1) and increased relative expression of *tnf*α (PC2) and likely reflect general immune activity and a shift towards innate responses, respectively. Overall assay expression was best predicted by a GLM composed of the variables REPRO, SSI and a one‐way interaction between SCHISTO_X and LENGTH. Within the model, sexually immature fish (REPRO = 1) had significantly higher expression of all genes relative to partially mature (REPRO = 2) or fully mature (REPRO = 3) individuals (Figure 1a). Additionally, overall immune gene expression declined significantly with increasing *S. solidus* infection intensity (SSI) (Figure 1b). Larger fish had significantly greater overall assay expression; however, infection with *S. solidus* inverted this relationship (Figure 1c).

**Table 3:** Model effects for minimum adequate GLMs fitted for the REIV & HOSTA analysis and LMMs fitted for the analysis of all lochs. “Overall Assay Expression” represents PC1 for both analyses and represents the correlated relative expression of all immune genes. “Relative *tnf*α Expression” represents PC2 for both analyses and represents increased relative expression of the proinflammatory gene *tnf*α

Analysis	PC	Factor	*F/*χ^2^ a	*df*	*p* b	Effect
REIV & HOSTA	Overall assay expression	Reproductive index	9.599	2,64	**<0.001**	NA
		Schistosomatic index	4.095	1,64	**0.047**	–
		Modified length :Schisto infection	11.205	1,64	**0.001**	NA
REIV & HOSTA	Relative *tnf*α expression	Loch	11.632	1,68	**0.001**	NA
		Breeding coloration	4.957	1,68	**0.029**	NA
All Lochs	Overall assay expression	Life history strategy	8.275	2,92	**0.016**	NA
All Lochs	Relative *tnf*α expression	Life history strategy	8.990	2,96	**0.011**	NA
		Schisto infection	9.288	1,96	**0.002**	NA

a *F* statistics were used for GLMs, χ^2^ for LMMs.

b Values in bold denote significance at <0.05 threshold.

The model selected for PC2 included the additive variables LOCH and RED. Fish sampled from loch REIV expressed the inflammatory gene *tnf*α more in comparison with the other immune genes assayed than did fish sampled from HOSTA (Figure 1d). Interestingly, fish that displayed breeding coloration were also found to express *tnf*α more in relation to their other immune genes than fish that were not displaying breeding coloration (Figure 1d).

Condition, approximated through ASI, varied with various factors. Crucially, sexually mature fish (REPRO = 3) were in significantly poorer condition than sexually immature (REPRO = 1) (Tukey, *p *<* *0.001) and partially mature individuals (REPRO = 2) (Tukey, *p *<* *0.001) (GLM, *F*_2,65_ = 9.957, *p *<* *0.001). Additionally, fish from REIV were in poorer condition than HOSTA fish (GLM, *F*_1,65_ = 5.715, *p *=* *0.020) and condition declined with age (GLM, *F*_1,65_ = 7.949, *p *=* *0.006).

#### 3.1.2 All lochs (Short‐lived vs. long‐lived)

**Figure 2:**
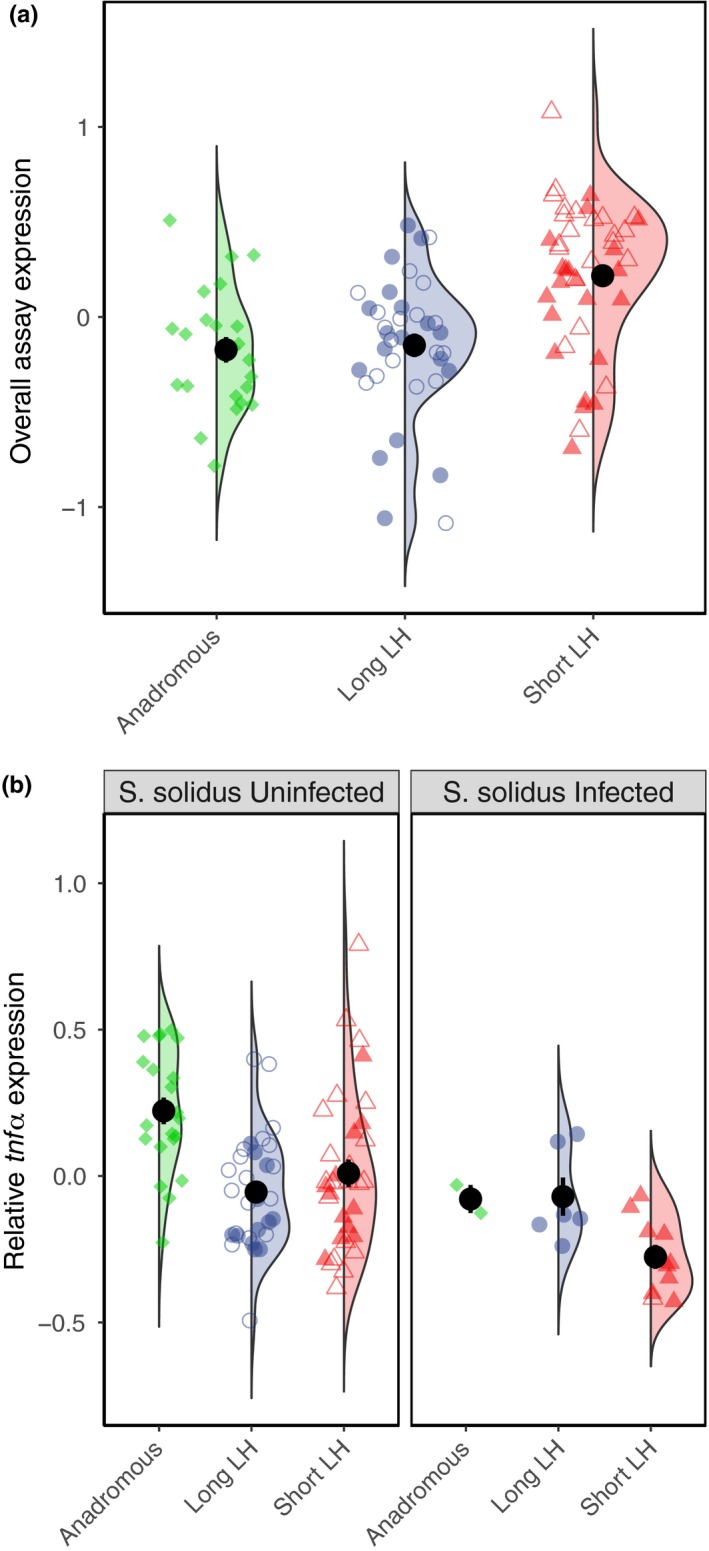
Significant factors in minimum adequate LMMs fitted to overall assay expression (PC1) scores (a) and relative *tnf*α expression (PC2) scores (b) for mature males sampled from all lochs. Half‐violins represent spread in the data, and central points denote group mean with standard error. Jittered points represent individual fish with shape representing life history strategy (circle = long‐lived, triangle = short‐lived, diamond = Anadromous and fill denoting population (long‐lived: filled = HOSTA, unfilled = REIV; short‐lived: filled = BHAR, unfilled = TORM). (a) Model effect plot for sampling location on expression of all immune genes. (b) The effect of sampling location and infection status with *S. solidus* on the increased relative expression of proinflammatory *tnf*α [Color figure can be viewed at http://www.wileyonlinelibrary.com]

For a summary of LMMs used, see Table 3. When comparing all mature fish across the short‐lived lochs TORM and BHAR, the long‐lived lochs REIV and HOSTA, and the ancestral marine fish OBSM, we found as above that PC1 (55.8%) represented the correlated expression of all immune genes and PC2 (21.5%) represented increased *tnf*α expression and reduced expression of the other four genes (Supporting Information Table S3; Figure S2). PC1 was best explained by a reduced model with LH as the only significant fixed effect (with LOCH retained as a random effect). Short‐lived populations exhibited the greatest expression of our assayed genes, with expression levels greater than anadromous fish (Tukey, *p *=* *0.055) and significantly greater than long‐lived fish (Tukey, *p *=* *0.026) (Figure 2a).

Increased relative *tnf*α expression was best predicted by an additive model including LH and SCHISTO_X as fixed effects and LOCH as a random effect. Anadromous fish had significantly higher relative expression of *tnf*α with respect to their other immune genes when compared to short‐lived (Tukey, *p *=* *0.009) and long‐lived (Tukey, *p *=* *0.009) freshwater strategists (Figure 2b). Furthermore, infection with *S. solidus* resulted in a reduction in relative expression of *tnf*α across the four populations in which it was found (Figure 2b), although numbers of infected fish were substantially lower in lochs OBSM (*N* = 2/23) and TORM (*N* = 1/22), compared with HOSTA (6/18) and BHAR (9/21).

To clarify that *Gyrodactylus* did not influence gene expression, expression variables PC1 and PC2 were modelled in *Gyrodactylus*‐infected fish from all lochs explicitly by *Gyrodactylus* burden. Neither expression variable was significantly associated with *Gyrodactylus* burden in infected fish (GLMs; PC1, *F*_1,50_ = 0.501, *p *=* *0.482; PC2, *F*_1,50_ = 2.677, *p *=* *0.108).

### 3.2 RAD‐seq analysis

#### 3.2.1 Population structure

**Figure 3:**
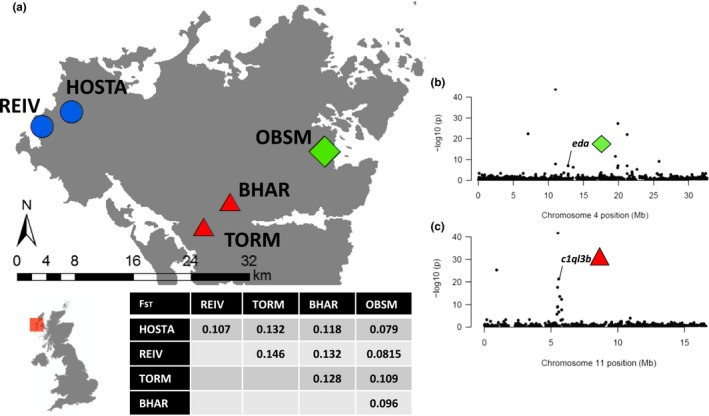
Genetic differentiation between lochs and life history strategies. (a) visualizes sampling locations and life history strategies for the five lochs sampled with accompanying genomewide pairwise *F*_ST_ scores. Shape and colour of points denote life history strategy; green (diamond) represents the anadromous population, blue (circle) represents long‐lived populations, and red (triangle) represents short‐lived populations. (b) Genetic differentiation scores for chromosome IV SNPs, plotted as –log10 *p*‐values of *F*_ST_ scores given by arlequin. The *eda* gene is highlighted as having potential associations with *tnf*α and is fixed in anadromous and freshwater comparisons. (c) Genetic differentiation scores for chromosome XI SNPs, plotted as –log10 *p*‐values of *F*_ST_ scores given by arlequin. The *c1ql3b* gene is highlighted as having potential roles within the complement system and has variants strongly associated with either short‐lived or long‐lived populations, with the long‐lived variant fixed in the anadromous fish [Color figure can be viewed at http://www.wileyonlinelibrary.com]

faststructure indicated a *k* value of 5, suggesting that each of our 5 lochs harbours a genetically distinct population of stickleback. As *k* increased from 1 to 5, it revealed an initial long‐lived, short‐lived divide at *k *=* *2, and at *k *=* *4, we had four distinct freshwater populations with remnants of all four freshwater populations found in each individual from the ancestral anadromous population. Pairwise *F*_ST_ values calculated from populations in STACKS confirm this structuring (Figure 3a).

#### 3.2.2 Outlier analysis

Of the 11,558 SNPs included in the analysis, arlequin highlighted 82 SNPs to be under significant selection. All 82 of these SNPs had higher than expected *F*_ST_ values and can therefore be assumed to be under directional selection as opposed to balancing selection. Of these 82 SNPs, 79 were also found to be in the 95th percentile of X^T^X values yielding an error rate of 3.66% for our analysis. When mapped back to genes, the roles of these SNPs varied considerably, with genes associated with bone development, fin morphogenesis, calcium and other metal ion management, protein binding and membrane processes (Supporting Information Table S4). Two genes have probable associations with immune processes, *eda* and *c1ql3b*, which are involved in tumour necrosis factor binding and the complement arm of the innate response, respectively. Variants associated with *eda* on chromosome IV (Figure 3b) were fixed in the anadromous OBSM fish and fixed for the alternative in all freshwater populations. Variants of *c1ql3b* on chromosome XI (Figure 3c) were strongly associated with either the short‐lived populations TORM and BHAR or longer‐lived REIV and HOSTA, with the REIV/HOSTA major allele fixed within the ancestral OBSM.

#### 3.2.3 Genetic variance of assay genes

**Figure 4:**
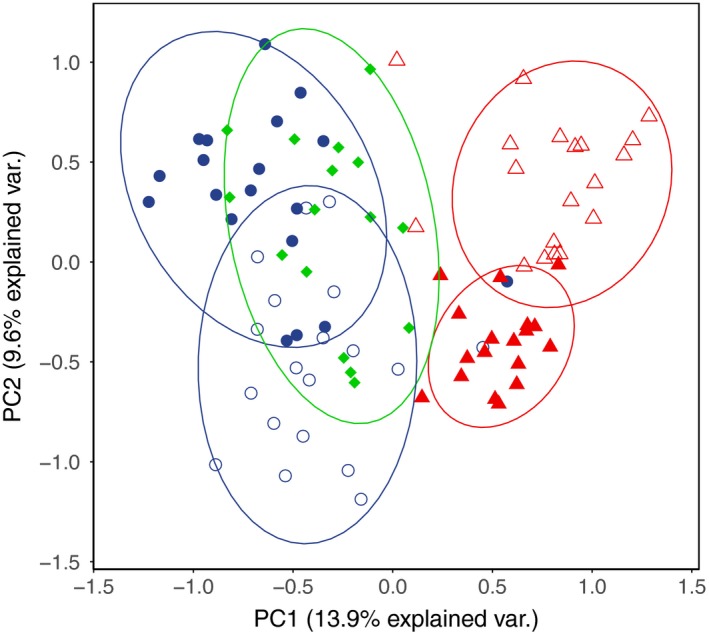
PCA for genetic variability of 51 SNPs located within 18 local (±50 kb) RAD‐loci in and around our five assayed immune genes. PC1 represents 13.9% of the total variability within the data, and PC2 represents 9.6% of the variability, giving a combined explanatory power of 25.5% for these two axes. Shape and colour of points denote life history strategy; green (diamond) represents the anadromous population, blue (circle) represents long‐lived populations, and red (triangle) represents short‐lived populations. Colour fill of points (filled = BHAR and HOSTA, unfilled = TORM and REIV) denotes populations. Ellipses represent 90% confidence intervals, which fully separate short‐lived populations from the other 3 [Color figure can be viewed at http://www.wileyonlinelibrary.com]

A PCA for genotypic variance in the 18 loci associated with our five immune assay genes between individuals reveals population separation within genotypic space. PC1, which represents 13.9% of the total variation, completely separates long‐lived and ancestral fish from short‐lived populations at the 90% confidence interval (Figure 4). This axis was driven predominantly by genetic variability in the regulatory gene *foxp3a* and the Th1‐activator *stat4*. Short‐lived populations were predominantly heterozygous (TORM = 0.559, BHAR = 0.385) for a T/C variant found in *foxp3a* (chrXII:16894963), whereas long‐lived and ancestral populations had elevated levels of homozygosity for the T variant (REIV = 0.833, HOSTA = 0.947, OBSM = 1.0). Short‐lived populations also had high levels of homozygosity for 2 SNPs found in stat4: the C variant of a C/G SNP (chrXVI:6372531) (TORM = 0.947, BHAR = 0.917) (REIV = 0.526, HOSTA = 0.474, OBSM = 0.633) and the A variant of an A/G SNP (chrXVI:6418158) (TORM = 0.868, BHAR = 0.972) (REIV = 0.553, HOSTA = 0.184, OBSM = 0.281).

There was slight population separation on PC2 which represented 9.6% of the variation (Figure 4). Separation on this axis was driven by genetic variability in *foxp3a*,* stat4* and the Th2‐activator *cmip*. Lochs BHAR, HOSTA and REIV had high levels of homozygosity for two SNPs found in *foxp3a*: the G variant of a G/A SNP (chrXII:16925720) (BHAR =  0.972, HOSTA = 0.974, REIV = 0.974) and the C variant of a C/T SNP (chrXII:16925570) (BHAR = 0.972, HOSTA = 0.947, REIV = 0.974). In *stat4,* the short‐lived populations showed high levels of homozygosity for the A variant of an A/G SNP (chrXVI:6418158) (TORM = 0.868, BHAR = 0.972), whilst in *cmip*, TORM fish were fixed for the A variant of an A/G SNP (chrXIX:1516826).

Taken together, these results suggest that short‐lived populations have diverged most significantly from long‐lived and ancestral fish predominantly in the genes *foxp3a* and *stat4*, whilst the additional divergence of *cmip* serves to partially separate TORM from other lochs in genotypic space.

**Figure 5:**
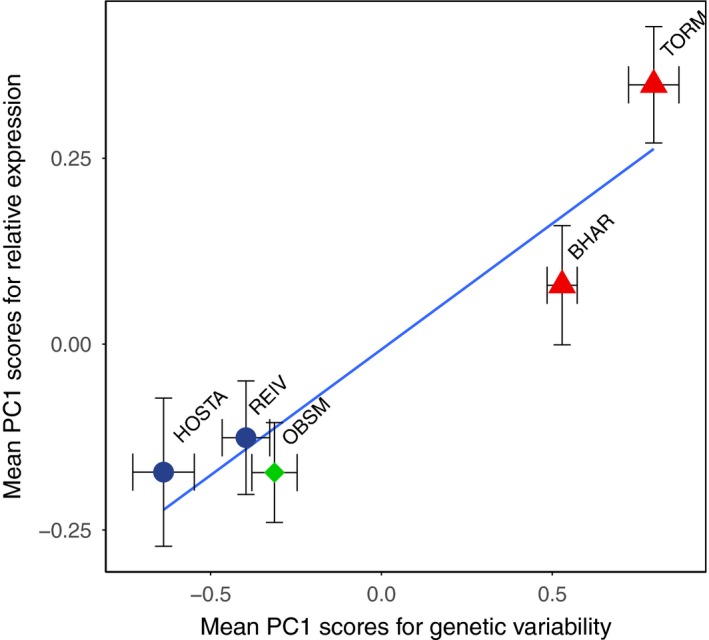
Association between population means for PC1 of relative expression (expression component scores) and PC1 of genetic variability of local SNPs with standard error represented along the *x*‐ and *y*‐axes. Population means were used as relative expression of immune genes was ascertained from fish sampled in this study's sampling effort (2015), whilst genetic variability was ascertained through RAD‐sequencing of fish from the same populations in a previous sampling effort (2013). The data show a strong association (*R* ^2^ = 0.943), which for the number of points present (*N* = 5) is likely substantial, despite an FDR‐corrected *p* value of 0.06 [Color figure can be viewed at http://www.wileyonlinelibrary.com]

When PC scores for SNP variability were averaged across populations and compared to average PC scores for expression, we found a correlation for SNP PC1 averages and expression PC1 averages (Pearson's, *R* ^2^ = 0.943, *p *=* *0.06) (Figure 5), which was just outside the 5% confidence interval after correcting for multiple comparisons. The strength of this relationship, given the small number of data points, suggests a potentially strong association between genetic variance in *foxp3a* and *stat4* between short‐lived and other populations and relative expression of all immune genes assayed. No other significant correlations were detected for other comparisons between SNP PC1, SNP PC2 and expression PC1 and expression PC2 (Pearson's, *p *=* *1.00).

### 3.3 Comparative relationship of immune gene genetic variance and life history

**Figure 6:**
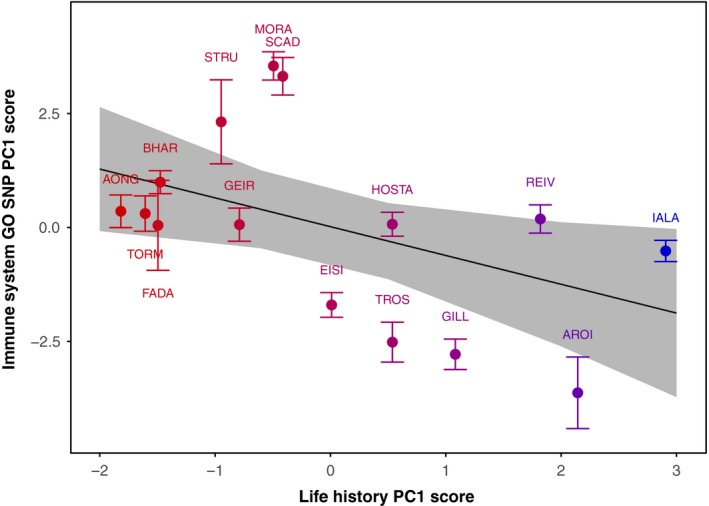
Linear regression between immune gene evolution and life history evolution across North Uist. Points denote population means ± standard deviation for immune gene genetic variation PC1 scores. Genetic variation PC1 scores are based on variation at 597 SNPs across 166 immune genes. Point colour gradient represents life history strategy denoted by Life history PC1 scores (red = short, blue = long). Life history PC1 scores are per population and represent common variation across: % wild individuals >1 year old; mean growth rate in 1 year; mean age at maturation; mean size at maturation. Fitted line represents mixed model predicted values with 95% confidence [Color figure can be viewed at http://www.wileyonlinelibrary.com]

PC1 for life history variation across the 15 populations represented 61.9% of the total variation and constituted the correlated variation of all 4 variables. There were significant linear relationships between genetic variation around immune genes (GO:0002376) (PC1 scores, PC1 = 13.4%) and life history PC1 scores (LMM, $\chi_{1,12}^{2}$ = 5.235, *p *=* *0.022) (Figure 6) and neutral structure (LMM, $\chi_{1,12}^{2}$ = 6.927, *p *=* *0.008). This relationship suggests that even when population structure is accounted for, populations with similar life history strategies are also more genetically similar in and around their immune genes.

## 4 DISCUSSION

We have documented consistent population‐level variation in immune gene expression associated with life history strategies. Further, this variation is associated with genetic variation around the genes assayed. There also exists a relationship across the North Uist radiation between genetic variation around a large number of immune genes and evolved life history strategies. Our data are suggestive that short life history strategies may be associated with increased immune activity, characterized by increased expression of five immunologically important genes alongside lower levels of observed parasitism. We also find that anadromous fish, which are semelparous with a short breeding season, exhibit more inflammatory expression profiles that may be characteristic of senescence (Franceschi, Garagnani, Vitale, Capri, & Salvioli, 2017; Franceschi et al., 2000).

Across life stages, we found that reproductive traits, such as maturation of testes and kidneys, and development of nuptial coloration, were associated with reduced overall expression of the assayed genes and increased relative *tnf*α expression, respectively. These observations, alongside our evidence that reproductive condition, defined by testes and kidney development, was associated with reduced body condition suggest that increased investment in reproductive traits may be traded off against aspects of immunity. These expression patterns were consistent across REIV and HOSTA, highlighting within‐population plasticity in expression and defining “cheaper” responses as those associated with poorer condition. Male stickleback tend to exhibit increased senescence and mortality towards the end of the breeding season in the wild (Dufresne, FitzGerald, & Lachance, 1990) and experimentally (Kim, Metcalfe, & Velando, 2016; Pike, Blount, Metcalfe, & Lindström, 2010), highlighting the stress associated with this period. There is agreement therefore in the patterns of increased relative *tnf*α between semelparous anadromous fish and individuals within populations exhibiting more sexually mature reproductive traits. Examination of raw relative expression ratios of individual genes suggests that in REIV and HOSTA, increased relative *tnf*α expression manifests through reduced expression of the other four genes, associated with adaptive and regulatory immune processes.

Data from wild skylarks, *Arlauda arvensis*, supports this hypothesis, as innate immunity is maintained through resource‐stressful periods (Hegemann, Matson, Versteegh, & Tieleman, 2012). Costs associated with downregulation of alternative immune processes, such as autoimmunity, may be offset by increased investment in other fitness‐boosting traits. During seasonal periods of resource scarcity and stress, typical of winter, humans exhibit increased expression of proinflammatory genes (Dopico et al., 2015), as do sticklebacks (Brown et al., 2016), which leads to seasonal bouts of autoimmune conditions such as arthritis in humans. Mechanistically, such seasonal plasticity may reflect modulation of immune responses by resource mediation of adipose tissue mass and, by association, concentrations of adipocytokines such as leptin, which is immunomodulatory, typically proinflammatory and suppresses regulatory Treg cell differentiation (La Cava & Matarese, 2004; Naylor & Petri, 2016). However, additional processes are probably involved, such as photoperiod‐induced changes to immunomodulatory thyroid hormones (Stevenson & Prendergast, 2015). Analogously, mammals trade off reproduction against somatic maintenance of oxidative stress during acute‐phase inflammation (Ashley, Weil, & Nelson, 2012). Predictably, effects are greatest in the most invested sex, such as female Soay sheep, *Ovis aries*, (Nussey, Pemberton, Pilkington, & Blount, 2009) and male Northern elephant seals, *Murounga angustirostris* (Sharick, Vazquez‐Medina, Ortiz, & Crocker, 2015).

Dependence on innate inflammatory responses in environmentally stressful conditions should reduce host condition and longevity, because of costs such as increased self‐damage and immunopathology (Dhinaut, Balourdet, Teixeira, Chogne, & Moret, 2017; Graham et al., 2005). However, these costs may be offset by immediate boosts to parasite immunity and other aspects of host fitness such as reproduction. According to theories of environmental ageing and senescence in the wild (Hayward, Wilson, Pilkington, Pemberton, & Kruuk, 2009), these immune costs may accelerate ageing. In the free‐ranging bat, *Saccopteryx bilineata*, mortality regimes across age classes are associated with immune parameters. Measures of response strength such as white blood cell counts and immunoglobulin G (IgG) concentrations decline in older age classes (Schneeberger, Courtiol, Czirják, & Voigt, 2014). In support, we observed that individuals in full reproductive condition displayed reduced condition (ASI), which also declined with age, reiterating the probable link between stickleback reproduction, its associated modification of immune responses and senescence.

Whilst we attribute increased relative *tnf*α expression to general reproductive investment, we cannot rule out associations with nuptial coloration directly. Here, immune changes may reflect direct associations with carotenoid allocation, which is important for ornamentation in stickleback (Wedekind, Meyer, Frischknecht, Niggli, & Pfander, 1998). Trade‐offs between sexual ornamentation and immune responses have been demonstrated in fighting fish, *Betta splendens*, (Clotfelter, Ardia, & McGraw, 2007) and guppies, *Poecilia reticulata* (Grether, Kasahara, Kolluru, & Cooper, 2004). In addition, supplementation of carotenoids in zebra finches, *Taeniopygia guttate*, alleviates trade‐offs and leads to redder sexual ornamentation and improved cellular and humoral immunity (Blount, Metcalfe, Birkhead, & Surai, 2003). In stickleback, low carotenoid diets are associated with high reproductive investment and increased senescence (Pike et al., 2010), strengthening the suggestion that increased relative *tnf*α expression is associated with senescence.

Parasitism by *S. solidus* was a prominent source of variation in both analyses, with infection resulting in suppression of immune genes in REIV and HOSTA, and relative *tnf*α expression in all lochs. This is to be expected given that *S. solidus* excretory products are suppressive (Scharsack, Gossens, Franke, & Kurtz, 2013), and the ability of *S. solidus* to modulate host immune responses is well documented (Barber & Scharsack, 2010; Scharsack, Kalbe, Derner, Kurtz, & Milinski, 2004; Scharsack, Koch, & Hammerschmidt, 2007). Infection with *G. arcuatus* had negligible effect on expression. This may reflect difficulties with assessing the stage of infections, which are very dynamic, such that a simple count of parasite abundance is a poor guide in wild fish. Immune gene expression is known to change over the course of *Gyrodactylus* infection (Lindenstrøm, Secombes, & Buchmann, 2004; Robertson, Bradley, & MacColl, 2017).

*Schistocephalus* and *Gyrodactylus* were included in our analyses because they are widespread and common and are likely to have effects on fitness. However, detailed studies of parasite communities on North Uist reveal that our short‐lived populations generally harbour less prevalent and less diverse parasite communities than our long‐lived populations (De Roij & MacColl, 2012; Rahn, Eßer, et al., 2016; Young & MacColl, 2017). This may stem from abiotic variation between populations, or differences in immunocompetence. Support for the former is weak (De Roij & MacColl, 2012; Rahn, Eßer, et al., 2016; Young & MacColl, 2017), but in support of the latter, in artificial infection experiments under laboratory conditions fish reared from our short‐lived population TORM display more resistance to *Gyrodactylus* than fish from our long‐lived population HOSTA (De Roij et al., 2011). However, it is probable given predictions of life history–immune evolution that environmental variation and immunocompetence are linked and therefore difficult to separate in nature.

Whilst we lack the functional data to confirm our interpretations, our expression data suggest that plasticity in sexually mature fish manifests as reduced overall assay expression (which may reflect general immune activity) and increased relative *tnf*α expression (shifts towards innate immunity) associated with reproductive traits, whilst parasitism further modulates expression. Lee (2006) predicts stressed individuals should plastically adopt cheaper responses, whilst short‐lived strategists should evolve costlier responses. Our data across populations supports this hypothesis, as short‐lived populations displayed increased overall assay expression, which was plastically associated with reduced reproductive condition and investment in the within‐population analysis. Whilst this may be environment‐driven, we sought to confirm that population‐level variation had an evolved genetic determinant. We observed a strong association between genetic variation in and around our five assay genes and their overall expression. Prominent SNPs were found in the Treg marker *foxp3a* and Th1‐adaptive marker *stat4*, with variants associated with short‐lived strategies displayed by fish from TORM and BHAR.

Divergence of Forkhead Box P3, encoded by *foxp3a*, is of particular interest given its role in the development and function of Treg cells. Treg cells are capable of suppressing other arms of the immune system (Long & Nanthakumar, 2004; Quintana et al., 2010), and increased Treg cell activation is further associated with a shift towards tolerant immune phenotypes (Dejaco, Duftner, & Schirmer, 2006). Tolerance represents a less costly alternative to resistant phenotypes (Downs, Adelman, & Demas, 2014; Hayward et al., 2014) and has been shown to be associated with older demographics of rodents in longer‐lived populations (Jackson et al., 2014). However, evidence to the contrary exists (Mayer, Brown, Zimmermann, & Shine, 2015) and the significance of Treg cells for tolerance in older age classes remains unclear (Nussey, Watt, Pilkington, Zamoyska, & McNeilly, 2012). In zebrafish, *foxp3a* is essential for immune tolerance, as the absence of a functional ortholog results in increased mortality through autoimmunity (Sugimoto, Hui, Sheng, Nakayama, & Kikuchi, 2017). Understanding the divergence of Treg processes and the importance of tolerance between diverging life history strategists represents a fruitful avenue for future research.

The relationship between overall assay expression and divergence at *stat4* is less clear. Associations with environmental variation between populations may explain this observation, given that our shorter‐lived populations live in more acidic conditions than the Western long‐lived populations (Magalhaes et al., 2016) and *stat4* resides in close proximity to the metal ion managing genes *adat3* and *fkbp7* (within 40 kb). Disentangling life history–immune associations from environmental variation is a challenge, but laboratory selection studies and sampling of other adaptive radiations with contrasting environmental patterns will aid in confirming relationships.

The use here of qPCR to sample mRNA levels of preselected genes of known importance facilitated biological replication necessary to elucidate patterns from noisy, wild data. Whilst the use of qPCR is generally common in ecoimmunology studies (reviewed by Fassbinder‐Orth, 2014), it is limited in scope when compared to other methods suitable for examining transcriptome‐level gene expression in nonmodels, such as RNA‐seq. As such, our ability to make functional inferences of the data is limited. As the costs of sequencing continue to decline alongside the increasing accessibility of high‐performance computing, the feasibility of combining transcriptome‐level analyses with high degrees of biological replication can build upon the data presented here. An additional potential limitation of all gene expression studies is the concern that mRNA levels associate poorly with post‐translational products of interest. Whilst early correlations between gene expression and protein levels were spurious, more recent analyses in mammals appear to confirm that gene expression is a dominant predictor of protein levels generally (Li, Bickel, & Biggin, 2014) and in immune‐specific contexts (Jovanovic et al., 2015).

At the genomic level, our outlier analysis revealed a SNP in the *c1ql3b* gene that showed strong divergence between short‐lived and all other populations. The *c1ql3b* gene encodes a q‐subcomponent of complement component 1 and may represent an active constituent of the fish innate inflammatory response (Ghai et al., 2007). In Japanese flounder, *Paralichthys olivaceus*,* c1ql3* is widely expressed in immune tissues including blood, liver and gills and recombinant C1ql3 has antimicrobial activity (Wang et al., 2017). However, *c1ql3* is predominantly expressed in the brain and in mammals has typically neurological function (NCBI Coordinators, N. R., 2018). Further, its role in active complement pathways is questionable, suggesting further evidence is required to demonstrate its immunological relevance. Divergence of complement responses may be in keeping with shorter life histories ameliorating the costs associated with innate and inflammatory processes, and indeed in garter snakes, complement responses are stronger in ecotypes that exhibit shorter life histories (Palacios, Sparkman, & Bronikowski, 2011), although these effects were not observed in all age classes.

By performing a comparative analysis across 15 populations, we provide evidence to show that populations that have evolved similar life history strategies have also evolved similar genetic variation around a large number (166) of immune genes across the genome. Population structure was controlled for in this analysis, and given strong population structure generally of stickleback on North Uist (Magalhaes et al., 2016; Rahn, Krassmann, Tsobanidis, MacColl, & Bakker, 2016), we can assume that each population represents an evolutionary replicate. Thus, the results documented in terms of difference in immune responses between life history strategies should be expected and should be expected to have an evolved genetic determinant. Further, this analysis demonstrates that life history evolution alone, irrespective of parasitism, is enough to explain evolutionary patterns of immune gene genetic variation. However, as previously discussed, it cannot be ruled out that parasitism communities are structured by environmental variation that also selects for life history strategy.

We found little evidence for genetic determinants of increased relative *tnf*α expression in our anadromous population. This suggests that variable expression patterns are encoded elsewhere in the genome or that this variation is plastic and driven by environmental associations. Addressing the first of these scenarios, our outlier analysis did reveal fixed SNPs between anadromous and freshwater populations around the *eda* gene. *eda* is a major adaptation gene repeatedly associated with freshwater adaptation and functionally involved with bony armour plate reduction (Colosimo, 2005; Jones, Grabherr, et al., 2012). The Eda protein harbours a tnf‐domain, suggesting it may be involved with inflammatory processes, although it appears more important for development in humans (Sadier, Viriot, Pantalacci, & Laudet, 2014). Other genes in close proximity, however, are likely to influence immunity and inflammation and are likely diverged between anadromous and freshwater populations given strong selection around *eda*. These genes include the B‐cell activating factor *tnfsf13b* (Baff) (Mackay & Browning, 2002), *garp* which interacts with *foxp3* and is involved in T‐cell function regulation (Probst‐Kepper & Buer, 2010), *dusp1* which controls inflammation (Hammer et al., 2010), and the mucin gene *muc5b* (El Nagar & MacColl, 2016; Jones, Chan, et al., 2012). Indeed, *eda* haplotypes in stickleback are associated with immune responses and parasite resistance when separated out from genetic background in F2 crosses (Robertson, Bradley, & MacColl, 2017). Additionally, marine stickleback raised in controlled conditions have more inflammatory responses to their own gut microbiota (Milligan‐Myhre et al., 2016), suggesting a genetic determinant for inflammatory variation.

Alternatively, anadromous fish may be under increased energetic stress having had to migrate inland to reproduce, a behaviour that predictably modifies life history (Snyder & Dingle, 1990). Once inland, anadromous stickleback must compete with diverged residents and may forego feeding to concentrate on reproduction, potentially intensifying resource‐associated immune plasticity. Few studies have examined the effect of migration on immune responses, although Eikenaar and Hegemann (2016) found innate immunity to be reduced in migratory blackbirds (*Turdus merula*), whilst Carbó‐Ramírez and Zuria (2015) found migratory species of sparrows to have stronger immune responses than nonmigrants. Contrasting patterns may occur as migration may either select for weaker immune responses to conserve energy, or stronger immunity to counteract a wider array of parasites.

In conclusion, we have been able to demonstrate consistent differences between the differential expression of immune genes between “short” and “long” life history strategies with a probable genetic determinant. Furthermore, by examining multiple populations, this study presents evidence with a degree of repeatability that is rarely available in the field of ecoimmunology. Disentangling plasticity and genetic determination is a challenge for ecoimmunology; however, we have attempted to address it here by examining within and between population variation. Within populations, we found differential expression patterns that are consistent with resource mediation hypotheses of immune variation, as stressed individuals downregulate overall assay expression and inflammatory‐moderating arms of the immune response. These patterns were observed at the population level as well between anadromous and fresh water residents, although we cannot rule out a genetic determinant for this. The similarities suggest a strong role for the environment in skewing the stickleback immune system to increased *tnf*α expression. Strong agreement between local allele frequencies and overall expression of our sampled genes, as well as divergence in a gene with possible complement function, seem to indicate that short‐lived populations may have diverged towards increased immune activity and costlier responses, a concept in keeping with current hypotheses. Finally, we reveal a general association across North Uist between life history trait evolution and genetic variation around immune genes across the genome, which confirms an evolutionary association between the two. Our findings thus highlight genuine evolutionary associations between life history and immune responses and their phenotypic prevalence in natural systems. Future research should take a more classical quantitative genetics approach, such as the use of common garden experiments, to tease apart the genetic underpinnings of this relationship. Towards this endeavour, the genes and responses highlighted may represent promising avenues of targeted research to further understand the interactions between life history and immune variation in the wild.

## DATA ACCESSIBILITY

Field sampling data, qPCR expression ratios and modelled PCs; faststructure input; arlequin input; bayenv input; and adegenet PCA analyses input: Dryad https://doi.org/10.5061/dryad.d9065.

Aligned reads for each individual have been deposited in the European Nucleotide Archive database (ENA Accession Number: PRJEB26681).

## CONFLICT OF INTEREST

The authors declare no conflict of interests.

## AUTHOR CONTRIBUTION

JRW and ADCM were involved in sampling design. JRW completed sampling, qPCR, data analysis, bioinformatics and writing. ISM performed RAD‐seq and was assisted in sampling of these fish in 2013 by DD. ARS studied life history across North Uist populations. SR designed and tested qPCR assays and assisted with RNA extractions. JEB and ADCM contributed towards the writing.
