# Supplementary material for: A genetics‐based approach confirms immune associations with life history across multiple populations of an aquatic vertebrate (Gasterosteus aculeatus)
Source: Mol Ecol. 2018 Jul 9;27(15):3174–91. doi: 10.1111/mec.14772 (PMC6221044; doi:10.1111/mec.14772)
Supplement: Supplementary file 1 [file MEC-27-3174-s001.pdf]

SUPPORTING INFORMATION

Supporting information 1

The effect of individual fish sampling order on gene expression was tested using a Pearson's correlation in the R package '*psych'* using 'holm' corrections for multiple testing. Sampling order of individual fish had no effect on relative expression of immune genes when studying all fish from REIV and HOSTA for PC1 (Pearson's, *r* = -0.13, *P* = 0.38) and PC2 (Pearson's, *r* = -0.01, *P* = 0.93) and all breeding fish for PC1 (Pearson's, *r* = -0.12, *P* = 0.63) and PC2 (Pearson's, *r* = -0.08, *P* = 0.63).

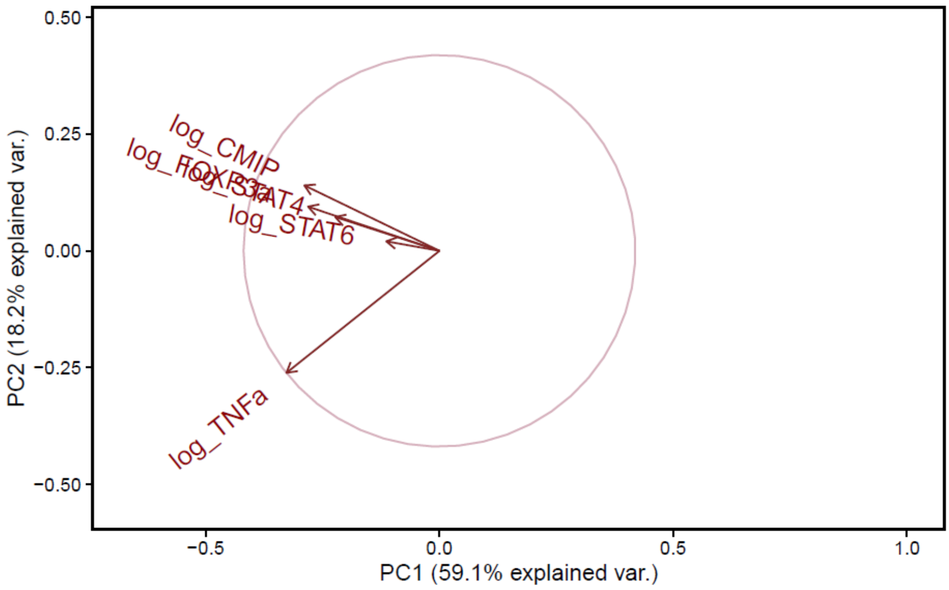

Figure S1 Principal Component Analysis demonstrating the covariance of relative expression of the 5 immune genes assayed for all fish from lochs REIV and HOSTA. Relative expression values were log-transformed prior to analysis. Eigenvectors show covariance for all immune genes along PC1 which represents 59.1% of the total variation. PC2 shows increasing expression of the pro-inflammatory gene *tnfα* relative to the other 4 genes, with this axis representing 18.2% of the overall variation. In total, the first 2 principal components camptured 77.3% of the variation in gene expression. PC scores were inverted for final analysis, so increasing PC scores corresponded to increased overall expression and increased *tnfα* expression for PCs 1 and 2 respectively.

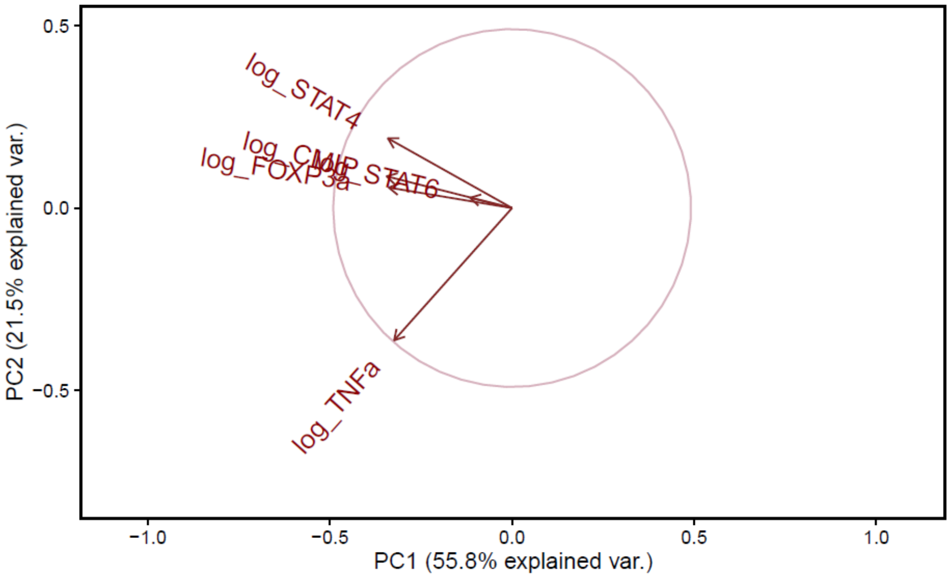

Figure S2 Principal Component Analysis demonstrating the covariance of relative expression of the 5 immune genes assayed for breeding males from all 5 lochs. Relative expression values were log-transformed prior to analysis. Eigenvectors show covariance for all immune genes along PC1 which represents 55.8% of the total variation. PC2 shows increasing expression of the pro-inflammatory gene *tnfα* relative to the other 4 genes, with this axis representing 21.5% of the overall variation. In total, the first 2 principal components camptured 77.3% of the variation in gene expression. PC scores were inverted for final analysis, so increasing PC scores corresponded to increased overall expression and increased *tnfα* expression for PCs 1 and 2 respectively.

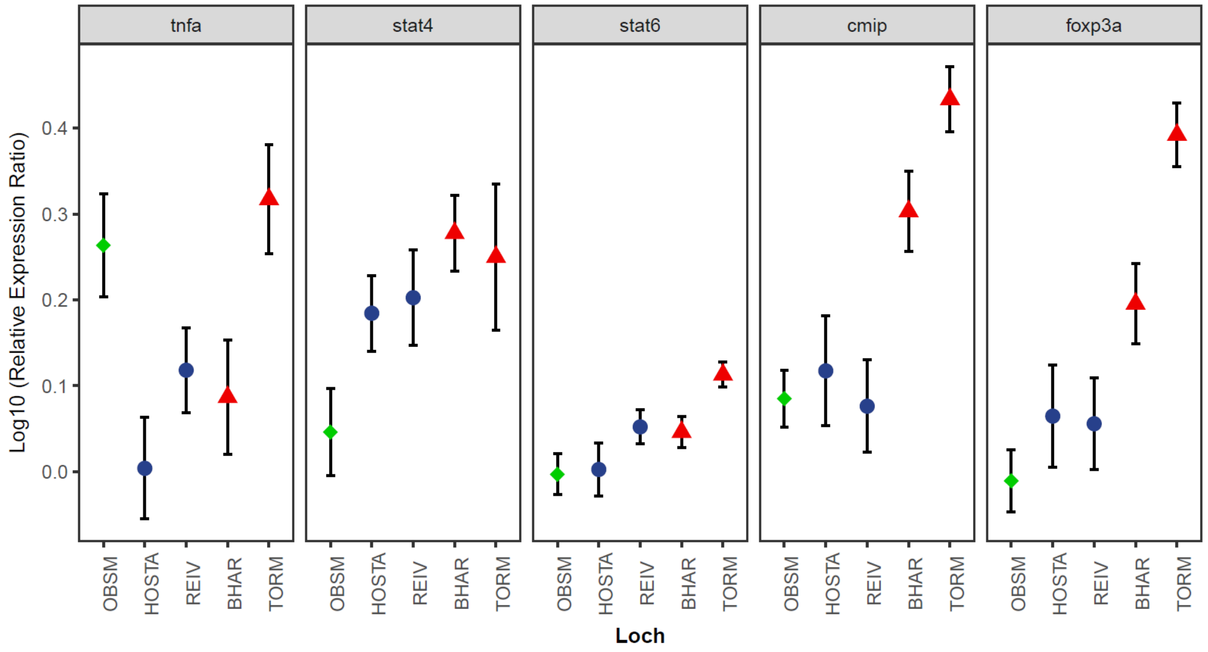

Figure S3 Log_{10}-transformed relative expression ratios for individual genes across adult males from the 5 sampled lochs. Values represent group means with standard errors. Point shapes and colours denote life history strategy ● = Long-lived, ▲ = Short-lived, ◆ = Anadromous.

Table S1: Sampling locations and life history information for lochs included in comparative analysis.

Loch	Latitude	Longitude	Older than 1 year (%)	Size at maturity (mm)	Absolute growth rate (mm year^{-1})	Age at maturity (Years)	Life History PC1
AONG	57°39"N	7°16"W	2.94	29.22	27.94	1.04	-1.82
AROI	57°35"N	7°25"W	8.82	48.41	41.99	1.40	2.14
BHAR	57°34"N	7°17"W	11.76	30.66	28.51	1.00	-1.48
EISI	57°38"N	7°21"W	14.71	36.08	31.87	1.30	0.01
FADA	57°36"N	7°12"W	5.88	30.22	27.75	1.13	-1.50
GEIR	57°38"N	7°17"W	17.14	32.02	29.50	1.16	-0.79
GILL	57°36"N	7°24"W	16.67	42.27	34.79	1.40	1.08
HOST	57°37"N	7°29"W	3.03	47.68	36.56	1.00	0.54
IALA	57°37"N	7°12"W	66.67	37.99	30.27	2.04	2.91
MORA	57°34"N	7°16"W	11.76	34.22	31.21	1.20	-0.49
REIV	57°37"N	7°31"W	27.27	46.84	34.92	1.43	1.82
SCAD	57°35"N	7°14"W	14.71	34.21	31.58	1.18	-0.41
STRU	57°34"N	7°21"W	8.82	33.91	31.70	1.00	-0.95
TORM	57°33"N	7°19"W	0.00	32.14	29.19	1.00	-1.61
TROS	57°35"N	7°25"W	3.03	43.46	38.62	1.06	0.54

Values are population means

Table S2: Location and descriptions of 166 immune genes for which genetic variation was captured in our comparative analysis. Information was extracted from Ensembl's BioMart.

Ensembl Gene ID	Associated Gene Name	Location	Gene Description
ENSGACG00000000027	mcm2	scaffold_89:278309-290469	minichromosome maintenance complex component 2 [Source:ZFIN;Acc:ZDB-GENE-020419-24]
ENSGACG00000000103	brd2a	scaffold_58:706703-713823	bromodomain containing 2a [Source:ZFIN;Acc:ZDB-GENE-990415-248]
ENSGACG00000000311	epc2	scaffold_69:762591-778555	enhancer of polycomb homolog 2 (Drosophila) [Source:ZFIN;Acc:ZDB-GENE-040426-802]
ENSGACG00000000330		scaffold_131:52153-54217	
ENSGACG00000000348		scaffold_131:118613-120538	
ENSGACG00000000350		scaffold_131:121399-126270	
ENSGACG00000000424		scaffold_27:865759-868515	
ENSGACG00000000433		scaffold_163:11092-22512	
ENSGACG00000000448	snrnp70	scaffold_80:215862-221505	small nuclear ribonucleoprotein 70 (U1) [Source:ZFIN;Acc:ZDB-GENE-040825-2]
ENSGACG00000000478	tnfa	scaffold_101:114081-115319	tumor necrosis factor a (TNF superfamily, member 2) [Source:ZFIN;Acc:ZDB-GENE-050317-1]
ENSGACG00000000657	wnt16	scaffold_90:335061-337313	wingless-type MMTV integration site family, member 16 [Source:ZFIN;Acc:ZDB-GENE-040426-2330]
ENSGACG00000000851	ash2l	scaffold_197:50068-59113	ash2 (absent, small, or homeotic)-like (Drosophila) [Source:ZFIN;Acc:ZDB-GENE-030131-494]
ENSGACG00000001162	bloc1s2	scaffold_218:24557-26120	biogenesis of lysosomal organelles complex-1, subunit 2 [Source:ZFIN;Acc:ZDB-GENE-050809-129]
ENSGACG00000001238	pdia5	scaffold_181:80653-94085	protein disulfide isomerase family A, member 5 [Source:ZFIN;Acc:ZDB-GENE-030521-5]
ENSGACG00000001275	cish	scaffold_27:3572123-3574221	cytokine inducible SH2-containing protein [Source:ZFIN;Acc:ZDB-GENE-050907-1]
ENSGACG00000001563	ddx18	groupXVI:1700437-1705166	DEAD (Asp-Glu-Ala-Asp) box polypeptide 18 [Source:ZFIN;Acc:ZDB-GENE-030131-9685]
ENSGACG00000001688	bmper	scaffold_122:1669-9217	BMP binding endothelial regulator [Source:ZFIN;Acc:ZDB-GENE-030219-146]
ENSGACG00000001692	eaf1	scaffold_122:11198-13232	ELL associated factor 1 [Source:ZFIN;Acc:ZDB-GENE-040625-109]
ENSGACG00000001729	cxcl8a	scaffold_882:1249-3529	chemokine (C-X-C motif) ligand 8a [Source:ZFIN;Acc:ZDB-GENE-081104-317]
ENSGACG00000001914	tcf7l1a	scaffold_176:97190-103179	transcription factor 7-like 1a (T-cell specific, HMG-box) [Source:ZFIN;Acc:ZDB-GENE-980605-30]
ENSGACG00000002010	ak2	groupX:1450596-1457347	adenylate kinase 2 [Source:ZFIN;Acc:ZDB-GENE-030131-512]
ENSGACG00000002163	cebp1	groupXXI:3041602-3042225	CCAAT/enhancer binding protein (C/EBP) 1 [Source:ZFIN;Acc:ZDB-GENE-010611-1]
ENSGACG00000002782	mta3	groupV:1856846-1875130	metastasis associated 1 family, member 3 [Source:ZFIN;Acc:ZDB-GENE-030131-6485]
ENSGACG00000003187	pdzk1ip1	groupVIII:1223738-1225334	PDZK1 interacting protein 1 [Source:ZFIN;Acc:ZDB-GENE-030616-128]
ENSGACG00000003451	pag1	groupX:4736748-4744791	phosphoprotein membrane anchor with glycosphingolipid microdomains 1 [Source:ZFIN;Acc:ZDB-GENE-060503-586]
ENSGACG00000003476	rps19	groupXX:819864-824313	ribosomal protein S19 [Source:ZFIN;Acc:ZDB-GENE-040426-1716]
ENSGACG00000003555	c1qc	groupXVII:821050-827904	complement component 1, q subcomponent, C chain [Source:ZFIN;Acc:ZDB-GENE-041010-51]

ENSGACG00000003574	rpl35	groupXIII:879549-881481	ribosomal protein L35 [Source:ZFIN;Acc:ZDB-GENE-020419-2]
ENSGACG00000003582		groupXVI:8023067-8024372	
ENSGACG00000003618		groupXIII:939506-944032	
ENSGACG00000003781	tgfbr2b	groupXX:1859763-1890241	transforming growth factor beta receptor 2b [Source:ZFIN;Acc:ZDB-GENE-980526-375]
ENSGACG00000003813	inpp5d	groupVIII:1936042-1944819	inositol polyphosphate-5-phosphatase D [Source:ZFIN;Acc:ZDB-GENE-100922-30]
ENSGACG00000003820	klhl6	groupXVI:8689838-8694756	kelch-like family member 6 [Source:ZFIN;Acc:ZDB-GENE-030616-325]
ENSGACG00000003871	arhgef3	groupXVII:1308549-1313794	Rho guanine nucleotide exchange factor (GEF) 3 [Source:ZFIN;Acc:ZDB-GENE-050208-773]
ENSGACG00000003968	tbpl2	groupXV:86505-89524	TATA box binding protein like 2 [Source:ZFIN;Acc:ZDB-GENE-040520-3]
ENSGACG00000003992	TLR8	groupXVI:8917742-8920801	toll like receptor 8 [Source:HGNC Symbol;Acc:HGNC:15632]
ENSGACG00000003996	tlr7	groupXVI:8922565-8926372	toll-like receptor 7 [Source:ZFIN;Acc:ZDB-GENE-040219-11]
ENSGACG00000004257	gfi1ab	groupVIII:2375426-2378608	growth factor independent 1A transcription repressor b [Source:ZFIN;Acc:ZDB-GENE-040116-8]
ENSGACG00000004323	traf3	groupXV:264877-279908	TNF receptor-associated factor 3 [Source:ZFIN;Acc:ZDB-GENE-040801-257]
ENSGACG00000004337	gpr1	groupXVI:9567216-9570039	G protein-coupled receptor 1 [Source:ZFIN;Acc:ZDB-GENE-091204-457]
ENSGACG00000004373	dkc1	groupXV:330050-334625	dyskeratosis congenita 1, dyskerin [Source:ZFIN;Acc:ZDB-GENE-031118-120]
ENSGACG00000004381	TLR5	groupXVIII:967664-972041	toll like receptor 5 [Source:HGNC Symbol;Acc:HGNC:11851]
ENSGACG00000004508	emilin1a	groupXVIII:1155689-1171739	elastin microfibril interfacer 1a [Source:ZFIN;Acc:ZDB-GENE-041001-191]
ENSGACG00000004570	arrb1	groupI:244666-253839	arrestin, beta 1 [Source:ZFIN;Acc:ZDB-GENE-060824-1]
ENSGACG00000004673	snx5	groupVI:5842529-5847391	sorting nexin 5 [Source:ZFIN;Acc:ZDB-GENE-040426-2857]
ENSGACG00000004684	mettl22	groupXI:294905-303815	methyltransferase like 22 [Source:ZFIN;Acc:ZDB-GENE-081022-70]
ENSGACG00000004688	plcg1	groupVIII:3202128-3218830	phospholipase C, gamma 1 [Source:ZFIN;Acc:ZDB-GENE-030421-3]
ENSGACG00000004727	fam49a	groupXVIII:1364627-1383998	family with sequence similarity 49, member A [Source:ZFIN;Acc:ZDB-GENE-031002-50]
ENSGACG00000004755	topbp1	groupXXI:10192181-10202166	topoisomerase (DNA) II binding protein 1 [Source:ZFIN;Acc:ZDB-GENE-060626-1]
ENSGACG00000004774	cdca7a	groupXVI:10081080-10084296	cell division cycle associated 7a [Source:ZFIN;Acc:ZDB-GENE-050417-29]
ENSGACG00000004823	brf1a	groupXV:972109-1008513	BRF1, RNA polymerase III transcription initiation factor a [Source:ZFIN;Acc:ZDB-GENE-030131-6334]
ENSGACG00000004857	ap2a1	groupXI:457068-477644	adaptor-related protein complex 2, alpha 1 subunit [Source:ZFIN;Acc:ZDB-GENE-090302-2]
ENSGACG00000004891		groupXV:1299495-1303130	
ENSGACG00000004892	tinagl1	groupX:7447952-7461974	tubulointerstitial nephritis antigen-like 1 [Source:ZFIN;Acc:ZDB-GENE-060503-240]
ENSGACG00000004913	glrx5	groupXVIII:1488667-1490560	glutaredoxin 5 homolog (S. cerevisiae) [Source:ZFIN;Acc:ZDB-GENE-040426-1957]
ENSGACG00000004950	trpm4a	groupXI:496809-523053	transient receptor potential cation channel, subfamily M, member 4a [Source:ZFIN;Acc:ZDB-GENE-090302-3]
ENSGACG00000005065	nfkbiaa	groupXVIII:1969540-1972310	nuclear factor of kappa light polypeptide gene enhancer in B-cells inhibitor, alpha a [Source:ZFIN;Acc:ZDB-GENE-040426-2227]
ENSGACG00000005363	numb	groupXV:1596272-1609737	numb homolog (Drosophila) [Source:ZFIN;Acc:ZDB-GENE-060422-1]

ENSGACG00000005449	tlr22	groupXXI:11300455-11304049	toll-like receptor 22 [Source:ZFIN;Acc:ZDB-GENE-040220-5]
ENSGACG00000005869	numbl	groupI:2075764-2085083	numb homolog (Drosophila)-like [Source:ZFIN;Acc:ZDB-GENE-051113-340]
ENSGACG00000005929	si:ch1073-280e3.1	groupI:2161683-2166868	si:ch1073-280e3.1 [Source:ZFIN;Acc:ZDB-GENE-110411-261]
ENSGACG00000006796	ago2	groupX:9491782-9499684	argonaute RISC catalytic component 2 [Source:ZFIN;Acc:ZDB-GENE-110606-6]
ENSGACG00000006905	c7a	groupXIII:5768682-5775531	complement component 7a [Source:ZFIN;Acc:ZDB-GENE-081104-392]
ENSGACG00000006921	cxcl12b	groupVI:8102677-8107213	chemokine (C-X-C motif) ligand 12b (stromal cell-derived factor 1) [Source:ZFIN;Acc:ZDB-GENE-030721-1]
ENSGACG00000007257	sufu	groupVI:8373107-8378306	suppressor of fused homolog (Drosophila) [Source:ZFIN;Acc:ZDB-GENE-030131-6223]
ENSGACG00000007321	prf1.5	groupXIII:6525599-6528384	perforin 1.5 [Source:ZFIN;Acc:ZDB-GENE-081105-3]
ENSGACG00000007333	hrh1	groupXVII:6062940-6064520	histamine receptor H1 [Source:ZFIN;Acc:ZDB-GENE-070531-3]
ENSGACG00000007505		groupVIII:8499419-8502408	
ENSGACG00000007901	si:ch211-173d10.1	groupV:9813451-9824646	si:ch211-173d10.1 [Source:ZFIN;Acc:ZDB-GENE-121214-115]
ENSGACG00000008392	aggf1	groupXVII:7882411-7887903	angiogenic factor with G patch and FHA domains 1 [Source:ZFIN;Acc:ZDB-GENE-060929-836]
ENSGACG00000008415	kalrna	groupXVI:16576938-16628180	kalirin RhoGEF kinase a [Source:ZFIN;Acc:ZDB-GENE-100921-4]
ENSGACG00000008529	med24	groupV:10604655-10612991	mediator complex subunit 24 [Source:ZFIN;Acc:ZDB-GENE-030131-2341]
ENSGACG00000008619	foxn1	groupI:7590810-7595653	forkhead box N1 [Source:ZFIN;Acc:ZDB-GENE-021008-1]
ENSGACG00000008634	stat5a	groupXI:5771316-5781666	signal transducer and activator of transcription 5a [Source:ZFIN;Acc:ZDB-GENE-030820-2]
ENSGACG00000008703	cpsf1	groupX:13121536-13131615	cleavage and polyadenylation specific factor 1 [Source:ZFIN;Acc:ZDB-GENE-040709-2]
ENSGACG00000008736	rps24	groupVI:9862404-9866201	ribosomal protein S24 [Source:ZFIN;Acc:ZDB-GENE-040109-5]
ENSGACG00000008796	myct1a	groupXVIII:8375481-8376824	myc target 1a [Source:ZFIN;Acc:ZDB-GENE-041001-143]
ENSGACG00000008839	sf3b1	groupXVI:17496458-17507672	splicing factor 3b, subunit 1 [Source:ZFIN;Acc:ZDB-GENE-040827-3]
ENSGACG00000008893	ube2ib	groupV:10838648-10842446	ubiquitin-conjugating enzyme E2Ib [Source:ZFIN;Acc:ZDB-GENE-990614-17]
ENSGACG00000009000	sod2	groupXVIII:8720471-8723389	superoxide dismutase 2, mitochondrial [Source:ZFIN;Acc:ZDB-GENE-030131-7742]
ENSGACG00000009364	tlr21	groupXX:10893595-10896378	toll-like receptor 21 [Source:ZFIN;Acc:ZDB-GENE-040220-4]
ENSGACG00000009452	cicb	groupXX:11098052-11122011	capicua transcriptional repressor b [Source:ZFIN;Acc:ZDB-GENE-030131-1966]
ENSGACG00000009662		groupX:14795391-14808405	
ENSGACG00000009679	kdm1a	groupXV:7938586-7949974	lysine (K)-specific demethylase 1a [Source:ZFIN;Acc:ZDB-GENE-030131-7828]
ENSGACG00000009688	enpp2 (1 of many)	groupX:14812602-14836398	ectonucleotide pyrophosphatase/phosphodiesterase 2 [Source:ZFIN;Acc:ZDB-GENE-040426-1156]
ENSGACG00000009747	tnnt2a	groupXII:12120260-12127679	troponin T type 2a (cardiac) [Source:ZFIN;Acc:ZDB-GENE-000626-1]
ENSGACG00000009820	lpar3	groupVIII:12552722-12556194	lysophosphatidic acid receptor 3 [Source:ZFIN;Acc:ZDB-GENE-120202-1]
ENSGACG00000009831	etv7	groupXII:12218917-12222496	ets variant 7 [Source:ZFIN;Acc:ZDB-GENE-070209-53]
ENSGACG00000010017	acvr1l	groupXV:8312082-8317879	activin A receptor, type I like [Source:ZFIN;Acc:ZDB-GENE-990415-9]
ENSGACG00000010111	ptpn6	groupXX:11683770-11695746	protein tyrosine phosphatase, non-receptor type 6 [Source:ZFIN;Acc:ZDB-GENE-030131-7513]
ENSGACG00000010128		groupXX:11699550-11705272	

ENSGACG00000010138	f2r	groupXIII:9878841-9880899	coagulation factor II (thrombin) receptor [Source:ZFIN;Acc:ZDB-GENE-060526-30]
ENSGACG00000010141		groupXIII:9880102-9881725	
ENSGACG00000010226	p2ry12	groupI:10334339-10336553	purinergic receptor P2Y, G-protein coupled, 12 [Source:ZFIN;Acc:ZDB-GENE-110208-4]
ENSGACG00000010274	gata4	groupXVIII:10178187-10183283	GATA binding protein 4 [Source:ZFIN;Acc:ZDB-GENE-980526-476]
ENSGACG00000010301	tnfsf14	groupXI:8127608-8129632	TNF superfamily member 14 [Source:ZFIN;Acc:ZDB-GENE-101109-3]
ENSGACG00000010334		groupX:15441448-15447087	
ENSGACG00000010609	ptk2bb	groupXVIII:10583092-10597725	protein tyrosine kinase 2 beta, b [Source:ZFIN;Acc:ZDB-GENE-020507-1]
ENSGACG00000010765	slc25a37	groupXIII:11861876-11868788	solute carrier family 25 (mitochondrial iron transporter), member 37 [Source:ZFIN;Acc:ZDB-GENE-031118-202]
ENSGACG00000011100	sptb	groupXV:10442747-10472910	spectrin, beta, erythrocytic [Source:ZFIN;Acc:ZDB-GENE-000906-1]
ENSGACG00000011155	cd40	groupXII:14765314-14769502	CD40 molecule, TNF receptor superfamily member 5 [Source:ZFIN;Acc:ZDB-GENE-090313-95]
ENSGACG00000011219	ccl25b	groupVIII:14292465-14293697	chemokine (C-C motif) ligand 25b [Source:ZFIN;Acc:ZDB-GENE-110222-2]
ENSGACG00000011338	pink1	groupXII:15125655-15129365	PTEN induced putative kinase 1 [Source:ZFIN;Acc:ZDB-GENE-041212-53]
ENSGACG00000011960	tbl3	groupXI:10923087-10934128	transducin (beta)-like 3 [Source:ZFIN;Acc:ZDB-GENE-041114-104]
ENSGACG00000012004	flvcr1	groupXVIII:12849956-12854145	feline leukemia virus subgroup C cellular receptor 1 [Source:ZFIN;Acc:ZDB-GENE-041014-359]
ENSGACG00000012342	sae1	groupI:15780868-15792802	SUMO1 activating enzyme subunit 1 [Source:ZFIN;Acc:ZDB-GENE-040625-21]
ENSGACG00000012356		groupXX:13545828-13546986	
ENSGACG00000012539	rpl22l1	scaffold_126:56068-57459	ribosomal protein L22-like 1 [Source:ZFIN;Acc:ZDB-GENE-060804-3]
ENSGACG00000012697	sbds	groupI:16493304-16495346	SBDS, ribosome maturation factor [Source:ZFIN;Acc:ZDB-GENE-040426-1116]
ENSGACG00000012706	tbx20	groupXX:14432325-14440386	T-box 20 [Source:ZFIN;Acc:ZDB-GENE-000427-7]
ENSGACG00000012833	mfn2	groupXII:16997938-17009439	mitofusin 2 [Source:ZFIN;Acc:ZDB-GENE-081105-44]
ENSGACG00000013037	brd2b	groupXX:15423966-15435054	bromodomain containing 2b [Source:ZFIN;Acc:ZDB-GENE-070220-1]
ENSGACG00000013150	ntrk1	groupXX:15652471-15673846	neurotrophic tyrosine kinase, receptor, type 1 [Source:ZFIN;Acc:ZDB-GENE-980526-118]
ENSGACG00000013530	hsp90ab1	groupXVIII:15800515-15805626	heat shock protein 90, alpha (cytosolic), class B member 1 [Source:ZFIN;Acc:ZDB-GENE-990415-95]
ENSGACG00000013656	cnr2	groupXX:16941429-16942391	cannabinoid receptor 2 [Source:ZFIN;Acc:ZDB-GENE-040702-7]
ENSGACG00000013677	erap1b	groupXIII:16842384-16848624	endoplasmic reticulum aminopeptidase 1b [Source:ZFIN;Acc:ZDB-GENE-040426-934]
ENSGACG00000014351	prkcbb	groupXI:14886837-14931008	protein kinase C, beta b [Source:ZFIN;Acc:ZDB-GENE-040426-1178]
ENSGACG00000014381	tgs1	groupIII:4031366-4040463	trimethylguanosine synthase 1 [Source:ZFIN;Acc:ZDB-GENE-070802-2]
ENSGACG00000014596		groupI:22327881-22330473	
ENSGACG00000014598		groupI:22332779-22351627	
ENSGACG00000014635	rpl27	groupXI:15703559-15706139	ribosomal protein L27 [Source:ZFIN;Acc:ZDB-GENE-030131-4343]
ENSGACG00000014788		groupXI:15918843-15920596	
ENSGACG00000014838	ptk2ba	groupI:22919039-22927647	protein tyrosine kinase 2 beta, a [Source:ZFIN;Acc:ZDB-GENE-080207-1]
ENSGACG00000014855	prg4a	groupIII:6006282-6010221	proteoglycan 4a [Source:ZFIN;Acc:ZDB-GENE-030131-8295]

ENSGACG00000014930	kat8	groupXI:16305215-16309455	K(lysine) acetyltransferase 8 [Source:ZFIN;Acc:ZDB-GENE-030131-7510]
ENSGACG00000014989	tmod2	groupII:5711386-5723520	tropomodulin 2 [Source:ZFIN;Acc:ZDB-GENE-040912-185]
ENSGACG00000015087	jam2a	groupI:25211638-25219873	junctional adhesion molecule 2a [Source:ZFIN;Acc:ZDB-GENE-031204-3]
ENSGACG00000015112		groupII:6073194-6074507	
ENSGACG00000015302	rasa3	groupII:7675747-7702944	RAS p21 protein activator 3 [Source:ZFIN;Acc:ZDB-GENE-090313-21]
ENSGACG00000015362	jmjd1cb	scaffold_48:1393797-1410996	jumonji domain containing 1Cb [Source:ZFIN;Acc:ZDB-GENE-121214-344]
ENSGACG00000015516	ephx2	groupI:27641497-27646007	epoxide hydrolase 2, cytoplasmic [Source:ZFIN;Acc:ZDB-GENE-041212-70]
ENSGACG00000015581	sumo1	groupI:27801691-27803793	small ubiquitin-like modifier 1 [Source:ZFIN;Acc:ZDB-GENE-040426-2186]
ENSGACG00000015674	gfi1aa	groupIII:8866707-8868989	growth factor independent 1A transcription repressor a [Source:ZFIN;Acc:ZDB-GENE-050522-534]
ENSGACG00000015944	kitb	groupIX:503930-512558	v-kit Hardy-Zuckerman 4 feline sarcoma viral oncogene homolog b [Source:ZFIN;Acc:ZDB-GENE-050916-2]
ENSGACG00000016110	melk	groupIX:1462605-1468408	maternal embryonic leucine zipper kinase [Source:ZFIN;Acc:ZDB-GENE-990603-5]
ENSGACG00000016122	rgs18	groupIII:9445520-9448756	regulator of G protein signaling 18 [Source:ZFIN;Acc:ZDB-GENE-061013-722]
ENSGACG00000016394	tpma	groupII:15107892-15115361	alpha-tropomyosin [Source:ZFIN;Acc:ZDB-GENE-990415-269]
ENSGACG00000016494	SPPL2A	groupII:15546599-15556143	signal peptide peptidase like 2A [Source:HGNC Symbol;Acc:HGNC:30227]
ENSGACG00000016706	npm1b	groupIV:3219707-3229632	nucleophosmin 1b (nucleolar phosphoprotein B23, numatrin) [Source:ZFIN;Acc:ZDB-GENE-080723-7]
ENSGACG00000016815	trim2a	groupIX:5364846-5375967	tripartite motif containing 2a [Source:ZFIN;Acc:ZDB-GENE-050327-99]
ENSGACG00000016874	tlr3	groupIX:5558990-5568796	toll-like receptor 3 [Source:ZFIN;Acc:ZDB-GENE-040219-7]
ENSGACG00000016946	m17	groupXIV:6493200-6494250	IL-6 subfamily cytokine M17 [Source:ZFIN;Acc:ZDB-GENE-060526-368]
ENSGACG00000016950	SUSD2	groupXIV:6510961-6517608	sushi domain containing 2 [Source:HGNC Symbol;Acc:HGNC:30667]
ENSGACG00000017113	cdx4	groupIV:5597628-5600423	caudal type homeobox 4 [Source:ZFIN;Acc:ZDB-GENE-980526-330]
ENSGACG00000017142	traf6	groupII:20808865-20813575	TNF receptor-associated factor 6 [Source:ZFIN;Acc:ZDB-GENE-030131-5735]
ENSGACG00000017257		groupIV:6166412-6167818	
ENSGACG00000017271	il-15	groupIX:7296176-7299702	interleukin-15 [Source:RefSeq peptide;Acc:NP_001254613]
ENSGACG00000017305		groupXIV:7304055-7314207	
ENSGACG00000017405	snapc3	groupIX:7894305-7897888	small nuclear RNA activating complex, polypeptide 3 [Source:ZFIN;Acc:ZDB-GENE-040426-971]
ENSGACG00000017431	wnt9a	groupIII:13988988-14002984	wingless-type MMTV integration site family, member 9A [Source:ZFIN;Acc:ZDB-GENE-060825-97]
ENSGACG00000017460	ak3	groupXIV:7934731-7938753	adenylate kinase 3 [Source:ZFIN;Acc:ZDB-GENE-040426-2142]
ENSGACG00000017958	tlr1	groupIV:10586230-10589632	toll-like receptor 1 [Source:ZFIN;Acc:ZDB-GENE-040220-1]
ENSGACG00000018016		groupIV:10953105-10958318	
ENSGACG00000018050		groupIV:11248317-11248905	
ENSGACG00000018216	cbx8b	groupIX:10487353-10490421	chromobox homolog 8b [Source:ZFIN;Acc:ZDB-GENE-050522-325]

ENSGACG00000018311	eda	groupIV:12800220-12810446	ectodysplasin A [Source:ZFIN;Acc:ZDB-GENE-050107-6]
ENSGACG00000018312		groupIV:12811602-12817008	
ENSGACG00000018374	tnip1	groupIV:13389317-13398176	TNFAIP3 interacting protein 1 [Source:ZFIN;Acc:ZDB-GENE-070112-1502]
ENSGACG00000018522	lrsam1	groupXIV:14580165-14593681	leucine rich repeat and sterile alpha motif containing 1 [Source:ZFIN;Acc:ZDB-GENE-060526-97]
ENSGACG00000018551	psmb10	groupXIV:14667512-14672885	proteasome subunit beta 10 [Source:ZFIN;Acc:ZDB-GENE-040718-278]
ENSGACG00000018663	tmem173	groupIV:16118651-16126776	transmembrane protein 173 [Source:ZFIN;Acc:ZDB-GENE-120921-1]
ENSGACG00000018669	tlr2	groupVII:521009-526172	toll-like receptor 2 [Source:ZFIN;Acc:ZDB-GENE-040219-6]
ENSGACG00000018747	crebbpa	groupIX:14306771-14340792	CREB binding protein a [Source:ZFIN;Acc:ZDB-GENE-050208-439]
ENSGACG00000018814	ano6	groupIV:17939827-17946536	anoctamin 6 [Source:ZFIN;Acc:ZDB-GENE-081104-64]
ENSGACG00000018821	waslb	groupIV:18005474-18019156	Wiskott-Aldrich syndrome-like b [Source:ZFIN;Acc:ZDB-GENE-040426-718]
ENSGACG00000018910	tfec	groupIV:18862829-18870435	transcription factor EC [Source:ZFIN;Acc:ZDB-GENE-041210-21]
ENSGACG00000019035	sirt7	groupIX:15207597-15210662	sirtuin 7 [Source:ZFIN;Acc:ZDB-GENE-050208-612]
ENSGACG00000019051		groupVII:2444693-2447881	
ENSGACG00000019078	cxcl19	groupVII:2503503-2505336	chemokine (C-X-C motif) ligand 19 [Source:ZFIN;Acc:ZDB-GENE-140708-2]
ENSGACG00000019081		groupVII:2506486-2507989	
ENSGACG00000019143	klf1	groupIX:15525577-15527832	Kruppel-like factor 1 (erythroid) [Source:ZFIN;Acc:ZDB-GENE-980526-55]
ENSGACG00000019186	pik3cg	groupIV:22364132-22374435	phosphatidylinositol-4,5-bisphosphate 3-kinase, catalytic subunit gamma [Source:ZFIN;Acc:ZDB-GENE-040426-2532]
ENSGACG00000019209	sec23b	groupIX:15877319-15885755	Sec23 homolog B, COPII coat complex component [Source:ZFIN;Acc:ZDB-GENE-030131-5479]
ENSGACG00000019213	slc7a7	groupVII:3039104-3046186	solute carrier family 7 (amino acid transporter light chain, y+L system), member 7 [Source:ZFIN;Acc:ZDB-GENE-051127-5]
ENSGACG00000019282	b2m	groupIV:23806218-23810016	beta-2-microglobulin [Source:ZFIN;Acc:ZDB-GENE-980526-88]
ENSGACG00000019296	slc25a38b	groupIX:16371698-16376549	solute carrier family 25, member 38b [Source:ZFIN;Acc:ZDB-GENE-110214-1]
ENSGACG00000019471	rbpms	groupVII:4888854-4910614	RNA binding protein with multiple splicing [Source:ZFIN;Acc:ZDB-GENE-161017-145]
ENSGACG00000019605	etnppl	groupVII:6668657-6676279	ethanolamine-phosphate phospho-lyase [Source:ZFIN;Acc:ZDB-GENE-040426-1133]
ENSGACG00000019663	wnt5b	groupIV:28482725-28536218	wingless-type MMTV integration site family, member 5b [Source:ZFIN;Acc:ZDB-GENE-980526-87]
ENSGACG00000019897	cecr1b	groupIV:31023275-31026361	cat eye syndrome chromosome region, candidate 1b [Source:ZFIN;Acc:ZDB-GENE-041210-77]
ENSGACG00000019898	dpy30	groupIX:19955745-19956532	dpy-30 histone methyltransferase complex regulatory subunit [Source:ZFIN;Acc:ZDB-GENE-040718-136]
ENSGACG00000019957	cd36	groupIV:31346641-31351154	CD36 molecule (thrombospondin receptor) [Source:ZFIN;Acc:ZDB-GENE-040718-55]
ENSGACG00000020059	metap2b	groupIV:32276697-32279732	methionyl aminopeptidase 2b [Source:ZFIN;Acc:ZDB-GENE-030131-2172]
ENSGACG00000020270	akap10	groupVII:15243568-15257029	A kinase (PRKA) anchor protein 10 [Source:ZFIN;Acc:ZDB-GENE-030131-2535]
ENSGACG00000020825	gemin5	groupVII:26196674-26206155	gem (nuclear organelle) associated protein 5 [Source:ZFIN;Acc:ZDB-GENE-031112-9]
ENSGACG00000020876	txndc15	groupVII:27201860-27205236	thioredoxin domain containing 15 [Source:ZFIN;Acc:ZDB-GENE-070615-36]

Table S3: PC loadings for log-transformed qPCR relative expression ratios for the five immune genes included in this study. Loadings are grouped according to the two analyses conducted.

	HOSTA & REIV		All Lochs	
Gene	PC1	PC2	PC1	PC2
tnfα	0.568	0.816	0.474	0.856
stat4	0.488	-0.295	0.499	-0.451
stat6	0.197	-0.064	0.168	-0.067
cmip	0.501	-0.438	0.504	-0.205
foxp3a	0.387	-0.226	0.495	-0.133

Table S4: Genes highlighted as having significantly diverged SNPs within them according to both ARLEQUIN and BAYENV. Gene Ontology (GO) is included and was inferred through ENSEMBL's BioMart.

ENSEMBL Gene ID	Associated Gene Name	Location	GO Term Name	GO Term Accession
ENSGACG00000002977	pik3r4	groupXX:158613-177234	protein phosphorylation	GO:0006468
			ATP binding	GO:0005524
			protein kinase activity	GO:0004672
ENSGACG00000002987	col6a4a	groupXX:182137-239263	extracellular space	GO:0005615
ENSGACG00000004716	si:dkeyp-120h9.1	groupX:7178557-7197519	integral component of membrane	GO:0016021
			membrane	GO:0016020
			amino acid transmembrane transport	GO:0003333
			amino acid transmembrane transporter activity	GO:0015171
			integral component of membrane	GO:0016021
			membrane	GO:0016020
			amino acid transmembrane transport	GO:0003333
			amino acid transmembrane transporter activity	GO:0015171
ENSGACG00000004808	rapgef4	groupXVI:10104986-10129378	intracellular	GO:0005622
			positive regulation of GTPase activity	GO:0043547
			intracellular signal transduction	GO:0035556
			insulin secretion	GO:0030073
			cAMP-mediated signaling	GO:0019933
			regulation of exocytosis	GO:0017157
			small GTPase mediated signal transduction	GO:0007264
			cAMP binding	GO:0030552
			guanyl-nucleotide exchange factor activity	GO:0005085
ENSGACG00000005143	col1a1a	groupXI:910083-929209	extracellular matrix structural constituent	GO:0005201
			proteinaceous extracellular matrix	GO:0005578
			extracellular matrix structural constituent	GO:0005201
			extracellular matrix structural constituent	GO:0005201
ENSGACG00000006496	si:zfos-1425h8.1	groupXVI:12486311-12492879	metal ion binding	GO:0046872
			nucleic acid binding	GO:0003676
ENSGACG00000006736	kazna	groupXII:8388213-8475879	*NA*	*NA*
ENSGACG00000007138	atp2b2	groupXVII:5912952-5961420	integral component of membrane	GO:0016021

membrane	GO:0016020
calcium ion transmembrane transport	GO:0070588
calcium ion transport	GO:0006816
ion transport	GO:0006811
transport	GO:0006810
metal ion binding	GO:0046872
hydrolase activity	GO:0016787
ATP binding	GO:0005524
calcium-transporting ATPase activity	GO:0005388
nucleotide binding	GO:0000166
integral component of membrane	GO:0016021
membrane	GO:0016020
calcium ion transmembrane transport	GO:0070588
calcium ion transport	GO:0006816
ion transport	GO:0006811
transport	GO:0006810
metal ion binding	GO:0046872
hydrolase activity	GO:0016787
ATP binding	GO:0005524
calcium-transporting ATPase activity	GO:0005388
nucleotide binding	GO:0000166
integral component of membrane	GO:0016021
membrane	GO:0016020
calcium ion transmembrane transport	GO:0070588
calcium ion transport	GO:0006816
ion transport	GO:0006811
transport	GO:0006810
metal ion binding	GO:0046872
hydrolase activity	GO:0016787
ATP binding	GO:0005524
calcium-transporting ATPase activity	GO:0005388
nucleotide binding	GO:0000166
integral component of membrane	GO:0016021
membrane	GO:0016020
calcium ion transmembrane transport	GO:0070588

			calcium ion transport	GO:0006816
			ion transport	GO:0006811
			transport	GO:0006810
			metal ion binding	GO:0046872
			hydrolase activity	GO:0016787
			ATP binding	GO:0005524
			calcium-transporting ATPase activity	GO:0005388
			nucleotide binding	GO:0000166
ENSGACG00000007609	kif21b	groupXVII:6623655-6658654	microtubule-based movement	GO:0007018
			microtubule binding	GO:0008017
			ATP binding	GO:0005524
			microtubule motor activity	GO:0003777
			nucleotide binding	GO:0000166
			microtubule-based movement	GO:0007018
			microtubule binding	GO:0008017
			ATP binding	GO:0005524
			microtubule motor activity	GO:0003777
			nucleotide binding	GO:0000166
ENSGACG00000008403	ABCA7	groupVIII:9608799-9628295	integral component of membrane	GO:0016021
			membrane	GO:0016020
			phagocytosis	GO:0006909
			transport	GO:0006810
			ATPase activity	GO:0016887
			ATP binding	GO:0005524
			transporter activity	GO:0005215
			nucleotide binding	GO:0000166
ENSGACG00000008483	cntnap1	groupXI:5489109-5498480	paranode region of axon	GO:0033270
			integral component of membrane	GO:0016021
			membrane	GO:0016020
			paranodal junction assembly	GO:0030913
ENSGACG00000008517	c1ql3b	groupXI:5561299-5572291	collagen trimer	GO:0005581
			extracellular region	GO:0005576
ENSGACG00000008622	mast3b	groupVIII:9756535-9773010	phosphorylation	GO:0016310
			protein phosphorylation	GO:0006468
			transferase activity	GO:0016740

			kinase activity	GO:0016301
			ATP binding	GO:0005524
			protein serine/threonine kinase activity	GO:0004674
			protein kinase activity	GO:0004672
			magnesium ion binding	GO:0000287
			nucleotide binding	GO:0000166
ENSGACG00000009588	ttll7	groupVIII:12379136-12431134	cellular protein modification process	GO:0006464
ENSGACG00000010269		groupXIII:10374806-10407594	*NA*	*NA*
ENSGACG00000012005	noxo1a	groupXI:10935172-10937751	cytoplasm	GO:0005737
			oxidation-reduction process	GO:0055114
			phosphatidylinositol binding	GO:0035091
			superoxide-generating NADPH oxidase activity	GO:0016175
ENSGACG00000013546		groupXVIII:15825271-15830242	*NA*	*NA*
ENSGACG00000014095		groupII:394800-412877	*NA*	*NA*
ENSGACG00000016718	brd7	groupII:17942081-17949437	*NA*	*NA*
ENSGACG00000018030		groupIV:11007288-11014249	guanylate cyclase activity	GO:0004383
			phosphorus-oxygen lyase activity	GO:0016849
			heme binding	GO:0020037
			cGMP biosynthetic process	GO:0006182
			cyclic nucleotide biosynthetic process	GO:0009190
			intracellular signal transduction	GO:0035556
			intracellular	GO:0005622
			guanylate cyclase activity	GO:0004383
			phosphorus-oxygen lyase activity	GO:0016849
			heme binding	GO:0020037
			cGMP biosynthetic process	GO:0006182
			cyclic nucleotide biosynthetic process	GO:0009190
			intracellular signal transduction	GO:0035556
			intracellular	GO:0005622
ENSGACG00000018125	zgc:92275	groupXIV:11343325-11350025	oxidoreductase activity	GO:0016491
			metal ion binding	GO:0046872
			iron-sulfur cluster binding	GO:0051536
			2 iron, 2 sulfur cluster binding	GO:0051537
			oxidation-reduction process	GO:0055114
ENSGACG00000018298	vma21	groupIV:12660845-12838539	vacuolar proton-transporting V-type ATPase complex assembly	GO:0070072

			endoplasmic reticulum	GO:0005783
			endoplasmic reticulum membrane	GO:0005789
			ER to Golgi transport vesicle membrane	GO:0012507
			membrane	GO:0016020
			integral component of membrane	GO:0016021
			cytoplasmic vesicle	GO:0031410
			endoplasmic reticulum-Golgi intermediate compartment membrane	GO:0033116
ENSGACG00000018311	eda	groupIV:12800220-12810446	tumor necrosis factor receptor binding	GO:0005164
			immune response	GO:0006955
			membrane	GO:0016020
			integral component of membrane	GO:0016021
			tumor necrosis factor receptor binding	GO:0005164
			immune response	GO:0006955
			membrane	GO:0016020
			integral component of membrane	GO:0016021
ENSGACG00000018391	ctnna1	groupIV:13527626-13591025	structural molecule activity	GO:0005198
			beta-catenin binding	GO:0008013
			cadherin binding	GO:0045296
			actin filament binding	GO:0051015
			actin filament organization	GO:0007015
			cell adhesion	GO:0007155
			epithelial cell-cell adhesion	GO:0090136
			cytosol	GO:0005829
			adherens junction	GO:0005912
			actin cytoskeleton	GO:0015629
ENSGACG00000018721	xpnpep1	groupIX:14153720-14165621	hydrolase activity	GO:0016787
			metal ion binding	GO:0046872
			metalloaminopeptidase activity	GO:0070006
			proteolysis	GO:0006508
			hydrolase activity	GO:0016787
			metal ion binding	GO:0046872
			metalloaminopeptidase activity	GO:0070006
			proteolysis	GO:0006508
ENSGACG00000018960	atxn10	groupIV:19908710-19916119	*NA*	*NA*

ENSGACG00000018964	FBLN1 (1 of many)	groupIV:19916813-19954054	calcium ion binding	GO:0005509
			peptidase activator activity	GO:0016504
			positive regulation of peptidase activity	GO:0010952
			extracellular matrix organization	GO:0030198
			extracellular region	GO:0005576
			proteinaceous extracellular matrix	GO:0005578
ENSGACG00000019066	slco1d1	groupIV:21221532-21246568	transporter activity	GO:0005215
			transport	GO:0006810
			ion transport	GO:0006811
			plasma membrane	GO:0005886
			membrane	GO:0016020
			integral component of membrane	GO:0016021
ENSGACG00000019134	RERG	groupIV:21897674-21925529	nucleotide binding	GO:0000166
			GTP binding	GO:0005525
			signal transduction	GO:0007165
			small GTPase mediated signal transduction	GO:0007264
			intracellular	GO:0005622
			membrane	GO:0016020
			nucleotide binding	GO:0000166
			GTP binding	GO:0005525
			signal transduction	GO:0007165
			small GTPase mediated signal transduction	GO:0007264
			intracellular	GO:0005622
			membrane	GO:0016020
ENSGACG00000019216	uvssa	groupIV:22626941-22670203	*NA*	*NA*
ENSGACG00000020773	ncam1a	groupVII:24387904-24556179	cell adhesion	GO:0007155
			neuron projection development	GO:0031175
			regulation of synaptic plasticity	GO:0048167
			membrane	GO:0016020
			integral component of membrane	GO:0016021

NA denotes an absence of Gene Ontology information for that gene within BioMart.
